# Structure-Activity Relationship Study of the Neuritogenic Potential of the Glycan of Starfish Ganglioside LLG-3 ‡

**DOI:** 10.3390/md13127062

**Published:** 2015-12-05

**Authors:** Megumi Yamagishi, Ritsuko Hosoda-Yabe, Hideki Tamai, Miku Konishi, Akihiro Imamura, Hideharu Ishida, Tomio Yabe, Hiromune Ando, Makoto Kiso

**Affiliations:** 1 Department of Applied Bioorganic Chemistry, Faculty of Applied Biological Sciences, Gifu University, 1-1 Yanagido, Gifu-shi, Gifu 501-1193, Japan; glyco_gf@gifu-u.ac.jp (M.Y.); h.tamai@tu-braunschweig.de (H.T.); konishi@gifu-u.ac.jp (M.K.); aimamura@gifu-u.ac.jp (A.I.); ishida@gifu-u.ac.jp (H.I.); kiso@gifu-u.ac.jp (M.K.); 2 Institute for Integrated Cell-Material Sciences (WPI-iCeMS), Kyoto University, Yoshida Ushinomiya-cho, Sakyo-ku, Kyoto 606-8501, Japan; ritsuko@gifu-u.ac.jp; 3 Department of Applied Life Science, Faculty of Applied Biological Sciences, Gifu University, 1-1 Yanagido, Gifu-shi, Gifu 501-1193, Japan

**Keywords:** ganglioside, starfish, neurite outgrowth, PC12 cell, SH-SY5Y cell, structure-activity relationship, MAPK/ERK signaling

## Abstract

LLG-3 is a ganglioside isolated from the starfish *Linchia laevigata*. To clarify the structure-activity relationship of the glycan of LLG-3 toward rat pheochromocytoma PC12 cells in the presence of nerve growth factor, a series of mono- to tetrasaccharide glycan derivatives were chemically synthesized and evaluated *in vitro*. The methyl group at C8 of the terminal sialic acid residue was crucial for neuritogenic activity, and the terminal trisaccharide moiety was the minimum active motif. Furthermore, the trisaccharide also stimulated neuritogenesis in human neuroblastoma SH-SY5Y cells via mitogen-activated protein kinase (MAPK) signaling. Phosphorylation of extracellular signal-regulated kinase (ERK) 1/2 was rapidly induced by adding 1 or 10 nM of the trisaccharide. The ratio of phosphorylated ERK to ERK reached a maximum 5 min after stimulation, and then decreased gradually. However, the trisaccharide did not induce significant Akt phosphorylation. These effects were abolished by pretreatment with the MAPK inhibitor U0126, which inhibits enzymes MEK1 and MEK2. In addition, U0126 inhibited the phosphorylation of ERK 1/2 in response to the trisaccharide dose-dependently. Therefore, we concluded that the trisaccharide promotes neurite extension in SH-SY5Y cells via MAPK/ERK signaling, not Akt signaling.

## 1. Introduction

Gangliosides, a complex family of sialylated glycosphingolipids, are abundant in the vertebrate nervous system and play an important role in the development of the central nervous system. There have been many reports indicating that gangliosides can induce neuronal differentiation. A ganglioside mixture extracted from bovine brain stimulated neurite outgrowth and neuronal differentiation of the SH-SY5Y cultured human neuroblastoma cell line [1,2]. SH-SY5Y cells differentiate into adrenergic, cholinergic, or dopaminergic neurons under stimulation by various differentiation-inducing factors such as retinoic acid, phorbol ester (12-*O*-tetradecanoylphorbol-13-acetate), platelet-derived growth factor (PDGF), brain-derived neurotrophic factor (BDNF), nerve growth factor (NGF), basic fibroblast growth factor, insulin-like growth factor (IGF), and dibutyryl cyclic adenosine monophosphate [3,4,5,6,7]. These differentiation-inducing factors stimulate phosphorylation of Akt and extracellular signal-regulated kinase (ERK) 1/2 during neuronal differentiation [8,9,10,11,12]. Gangliosides can modify the effects of growth factors by enhancing or inhibiting their actions [13]. Mammalian GM1 ganglioside enhances the effect of NGF by binding to Tropomyosin receptor kinase A/NGF receptors in rat pheochromocytoma PC12 cells [14]. The action of epidermal growth factor (EGF) is inhibited by GM3 ganglioside binding to EGF receptors [15,16], whereas GD1a ganglioside enhances it [17]. In many cases, these effects were observed only when micromolar concentrations of gangliosides were added to the cultured cells. Micromolar levels of exogenous gangliosides might affect membrane fluidity and stability by being incorporated into the membrane, thus interfering with receptors and signaling proteins localized in glycolipid-enriched and raft membrane microdomains [18,19,20]. However, there is evidence showing that just the oligosaccharide portion of gangliosides can evoke biological responses *in vitro*. Oligosaccharides derived from GT1b or GM2 gangliosides could activate calmodulin-dependent protein kinase II and protein kinase A, resulting in neurite elongation when they were applied to primary cultured neurons at nanomolar levels [18,21,22]. These reports strongly suggest the presence of specific glycoreceptors on the cell surface.

New classes of gangliosides have been found in echinoderms, such as sea cucumbers [23,24,25], starfish [26,27,28,29,30,31], sea urchins [32,33,34], and feather stars [35], and many of these echinodermatous gangliosides (EGs) also exhibited neuritogenic activity, some of which exceeded that of mammalian GM1 [36,37]. In contrast to mammalian gangliosides, EGs contain partially modified sialic acid residues and their unique oligomers. In addition, the lipid moieties of EGs are very diverse, and have characteristic structures that have never been seen in mammalian gangliosides. Previously, we have synthesized EGs and their glycan moieties [38,39,40,41,42,43,44,45], and we also demonstrated the neurite outgrowth potentiation of PC12 cells by synthetic EGs (LLG-3 [40], GAA-7 [44], and PNG-2A [45]) in the presence of NGF. In the present study, we identified the minimum active glycan structure of LLG-3, the neuritogenic activity of which was the second most potent among fifteen EGs investigated despite its rather simple structure [36,37]. Therefore, several fragment derivatives of LLG-3 were synthesized to perform a structure-activity relationship study, so that we could identify the precise sugar structure of the LLG-3 ganglioside that produces the neuritogenic effect on PC12 and SH-SY5Y cells. We also revealed the activation of mitogen-activated protein kinase (MAPK) signaling pathway.

## 2. Results and Discussion

### 2.1. Synthesis of LLG-3 Analogues

**Figure 1:**
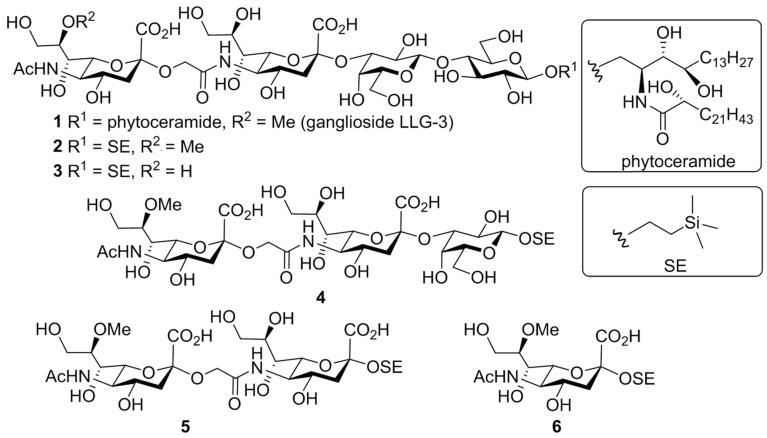
Structures of ganglioside LLG-3 and its analogues synthesized in this study.

To determine the minimum motif in LLG-3 (**1**) that potentiates neurite outgrowth of PC12 cells in the presence of NGF, a series of mono- to tetrasaccharide glycan derivatives were obtained by disconnecting the glycosidic bonds within LLG-3 (**1**) from the reducing end (**2**, **4**, **5**, and **6**) (Figure 1). In addition, demethylated tetrasaccharide derivative **3** was also designed to examine the effect of the methoxy group at the C8 position of the sialic acid residue on the neuritogenic activity.

**Scheme 1:**
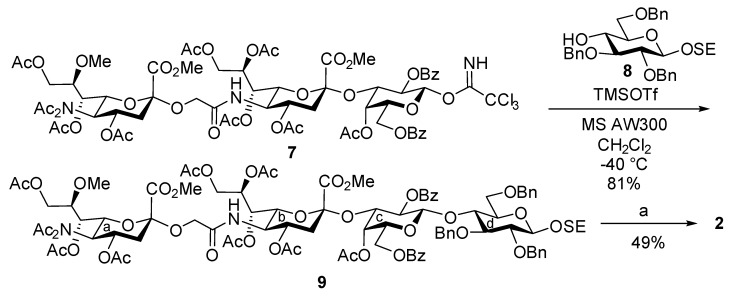
Synthesis of LLG-3 tetrasaccharide **2**. *Reagents and conditions*: (a) i. H_2_, Pd(OH)_2_-C/EtOAc, RT; ii. LiCl/Pyr, reflux; iii. 0.1 M NaOH aq., RT to 40 °C, 49% (3 steps).

The tetrasaccharide sequence in **2** was constructed by the glycosylation of glucosyl acceptor **8** [46] with trisaccharyl imidate donor **7**, which was developed in the previous study of the total synthesis of LLG-3 [40], under mild acidic conditions, giving tetrasaccharyl glycoside **9** in 81% yield (Scheme 1). Finally, global deprotection was performed as previously reported [47] to deliver LLG-3 tetrasaccharide **2**.

**Scheme 2:**
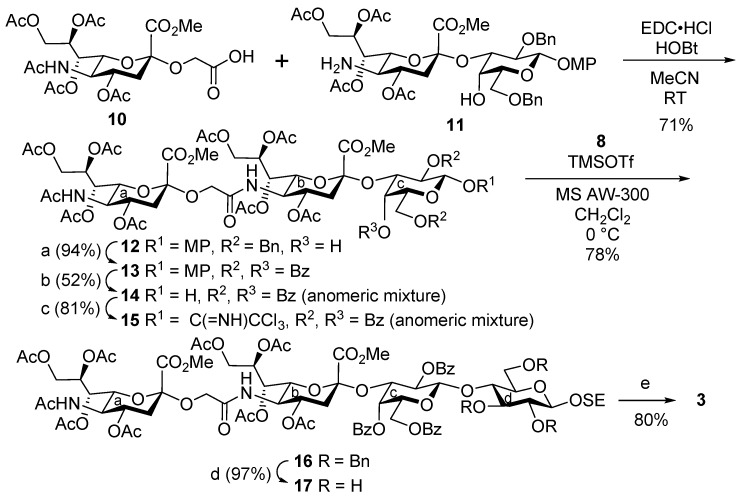
Synthesis of demethylated LLG-3 tetrasaccharide **3**. *Reagents and conditions*: (a) i. H_2_, Pd(OH)_2_-C, EtOAc, RT; ii. Bz_2_O, DMAP/Pyr, RT, 94% (2 steps); (b) i. CAN, toluene/MeCN/H_2_O (5/6/3), 0 °C; ii. Bz_2_O, DMAP/Pyr, RT; iii. NH_2_NH_2_·AcOH/DMF, RT, 52% (3 steps); (c) CCl_3_CN, DBU/CH_2_Cl_2_, 0 °C, 81%; (d) H_2_, Pd(OH)_2_-C/EtOAc, RT, 97%; (e) i. LiCl/Pyr, reflux; ii. 0.1 M NaOH aq., RT, 80% (2 steps). DMAP = 4-dimethylaminopyridine, CAN = cerium(IV) ammonium nitrate, DBU = 1,8-diazabicyclo[5.4.0]undec-7-ene.

To synthesize demethylated tetrasaccharide **3**, the outer trisaccharide moiety was constructed, and then it was combined with a glucose unit (Scheme 2). Thus, sialyl glycolic acid derivative **10** [48] was condensed with 5-amino-sialyl galactoside derivative **11** [40] in the presence of EDC·HCl and HOBt in MeCN to afford trisaccharide **12** in 71% yield. Next, trisaccharide **12** was converted to suitably protected glycosyl donor **15**, which was analogous to **7**, and **15** was then coupled with glucosyl acceptor **8**, producing tetrasaccharide **16** in 78% yield. After removal of the benzyl groups from **16**, a global deprotection procedure similar to that used for **9** delivered demethylated tetrasaccharide **3**.

**Scheme 3:**
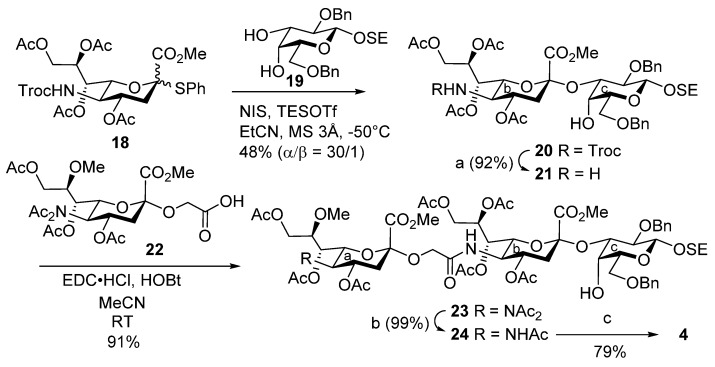
Synthesis of LLG-3 trisaccharide **4**. (a) Zn, AcOH/MeCN, RT, 92%; (b) NH_2_NH_2_·AcOH/THF, RT, 99%; (c) i. H_2_, Pd(OH)_2_-C/EtOAc, RT; ii. LiCl/Pyr, reflux; iii. 0.1 M NaOH aq., RT, 79% (3 steps). NIS = *N*-iodosuccinimide, TESOTf = triethylsilyl trifluoromethanesulfonate, EDC = 1-ethyl-3-(3-dimethylaminopropyl)carbodiimide, HOBt = 1-hydroxybenzotriazole.

For the synthesis of trisaccharide **4**, we attempted to obtain 2-(trimethylsilyl)ethyl (SE) glycoside by glycosidating trisaccharyl donor **7** with SE-OH under conventional reaction conditions (Scheme 3). However, the small amount of the stereoisomer (α-glycoside) that was also generated during the reaction showed similar mobility to the desired β-glycoside by TLC analysis, and the isomers could not be separated by chromatographic methods. To circumvent this problem, in the synthetic route in Scheme 3 suitably protected galactose **19** [49], which contained the β-SE glycoside, was used as the glycosyl acceptor in the glycosidation of sialic acid donor **18** [50], and disaccharide **20** was obtained in moderate yield. Next, selective removal of the Troc group with zinc and AcOH in MeCN and the ensuing coupling reaction using 8-Me-sialyl glycolic acid **22** [40] produced protected trisaccharide **23** in high yield. Finally, trisaccharide **23** underwent stepwise deprotection, including de-*N*-acetylation, demethylation, and basic ester hydrolysis, to afford **4**.

**Scheme 4:**
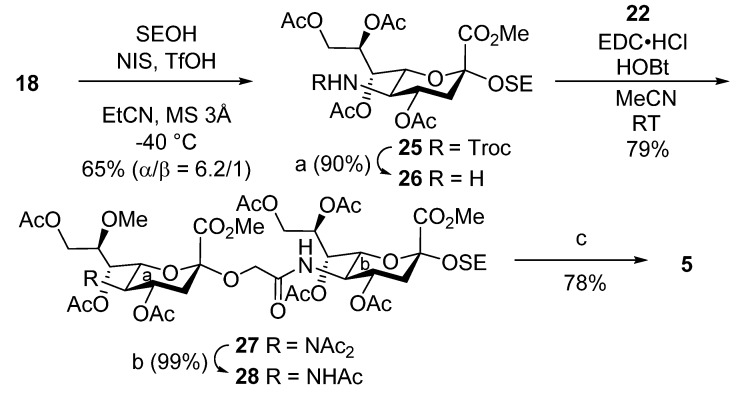
Synthesis of LLG-3 disaccharide **5**. (a) Zn, AcOH/MeCN, RT, 90%; (b) NH_2_NH_2_·AcOH/THF, RT, 99%; (c) i. LiCl/Pyr, reflux; ii. 0.1 M NaOH aq., RT, 78% (2 steps).

The synthesis of disaccharide **5** started with the glycosidation of sialyl donor **18** with SE-OH in the presence of NIS and TfOH [51] at −40 °C in EtCN, which was used as a stereo-directing reaction media [52] (Scheme 4). This reaction produced α-SE glycoside **25** with high stereoselectivity (α/β = 6.2/1), which was then converted to the amine derivative and condensed with **22** to afford protected disaccharide **27**. Finally, **27** was treated in a similar way to trisaccharide **23**, furnishing target compound **5**.

**Scheme 5:**
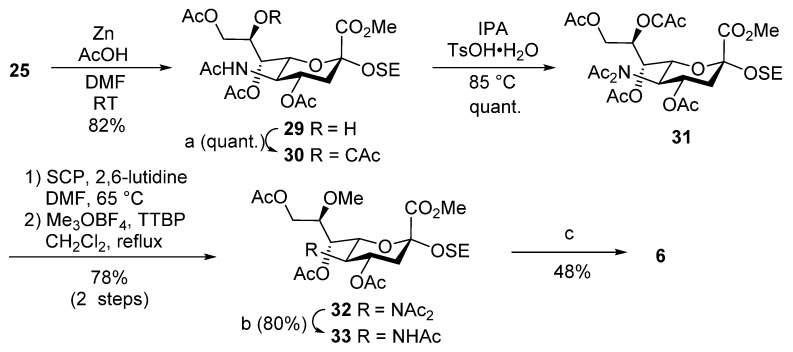
Synthesis of LLG-3 monosaccharide **6**. (a) CAc_2_O, DMAP/THF, RT, quant.; (b) NH_2_NH_2_·AcOH/THF, RT, 80%; (c) i. LiCl/Pyr, reflux; ii. 0.1 M NaOH aq., RT, 48% (2 steps). CAc = chloroacetyl, IPA = isopropenyl acetate, Ts = *p*-toluenesulfonyl, SCP = 1-selenocarbamoylpiperidine, TTBP = 2,4,6-tri-*tert*-butylpyrimidine.

In the final part of the syntheses of the LLG-3 glycan analogues, sialyl glycoside **25** was transformed into target compound **6** based on our method for the synthesis of 8-*O*-methyl sialic acid-containing molecules [40] (Scheme 5). First, **25** was converted into 8-OH derivative **29** via regioselective 8*O* to 5*N* migration of the acetyl group upon treatment with zinc under acidic conditions. Then, 8-OH protection with the chloroacetyl group gave **30**, and it was further modified to diacetylimide **31** by reaction with isopropenyl acetate in the presence of acid. Next, the chloroacetyl group was selectively cleaved by selenocarbamoylpiperidine [53], and the retrieved OH was methylated by Meerwein’s reagent, giving **32** in 78% yield over two steps. Finally, global deprotection produced monosaccharide **6**.

### 2.2. Neuritogenic Activity Evaluation

#### 2.2.1. Neurite Outgrowth Evaluation in PC12

**Figure 2:**
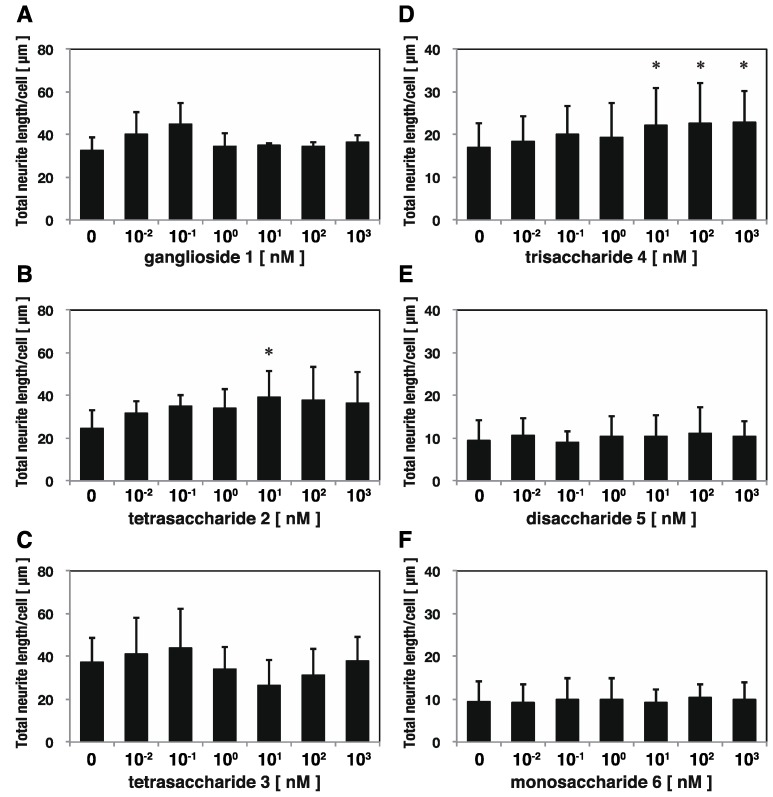
Neurite outgrowth evaluation in PC12 cells. Rat PC12 cells with a low serum culture medium containing 5 ng/mL of NGF were incubated with ganglioside **1** (**A**); tetrasaccharide **2** (**B**); tetrasaccharide **3** (**C**); trisaccharide **4** (**D**); disaccharide **5** (**E**) and monosaccharide **6** (**F**) for neurite outgrowth evaluation. The error bar represents the standard deviation (S.D.). * *p* < 0.05 with Dunnett’s test compared with the 0 nM group.

To evaluate the neuritogenic activity of the glycan moiety of LLG-3, the mean total neurite lengths per cell were measured in rat PC12 cells (Figure 2). Although LLG-3 tetrasaccharide **2** showed activity after 10 nM addition of 5 ng/mL NGF, tetrasaccharide **3** did not (Figure 2B,C). This result clearly indicates that the methoxy group at the C8 position of sialic acid residue affects the neuritogenic activity. Furthermore, to evaluate the minimum length of the glycan moiety of LLG-3 for neuritogenic activity, trisaccharide **4**, disaccharide **5**, and monosaccharide **6** were compared (Figure 2). Disaccharide **5** and monosaccharide **6** showed no neurite growth activity (Figure 2E,F). However, trisaccharide **4** induced substantial neuritogenic activity (Figure 2D), suggesting that the trisaccharide is the minimum essential glycan moiety of LLG-3 for neuritogenic activity in PC-12 cells. 

#### 2.2.2. Trisaccharide **4** Stimulated Neurite Extension in SH-SY5Y cells

**Figure 3:**
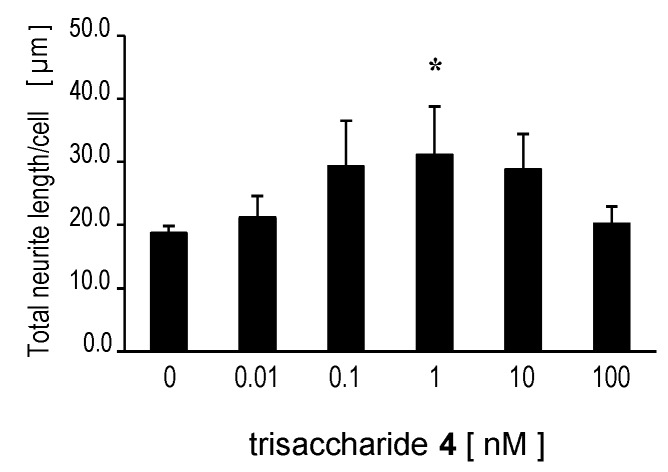
Trisaccharide **4**-induced neurite outgrowth in SH-SY5Y cells. Neuroblastoma SH-SY5Y cells were incubated with trisaccharide **4** for 3 days in medium containing 1% FBS and the mean total neurite length per cell calculated from 90 cells was measured at each dose. Trisaccharide **4** stimulated neurite extensions of SH-SY5Y cells in a dose-dependent manner. The error bar represents the standard deviation (S.D.). * *p* < 0.05 with Dunnett’s test compared with the 0 nM group.

We also examined the neuritogenic activity of trisaccharide **4** in human neuroblastoma SH-SY5Y cells. Trisaccharide **4** elongated SH-SY5Y neurites cultured in low serum-containing medium. The maximum neurite length was attained when 1 nM of trisaccharide **4** was added to the cells and the increase was statistically significant (*p* < 0.05, Dunnett’s test) (Figure 3). The neurite length increased up to 1 nM of trisaccharide **4** in a dose-dependent manner, and then decreased at higher concentrations (Figure 3).

#### 2.2.3. Activation of ERK Signaling in Response to Trisaccharide **4**

**Figure 4:**
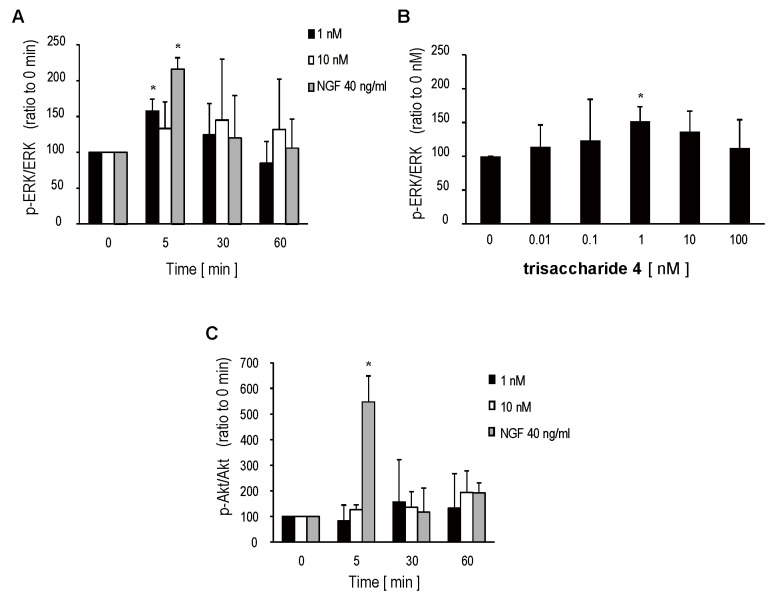
Effect of trisaccharide **4** on phosphorylation of p44/42 MAPK (ERK 1/2) and Akt. SH-SY5Y cells were incubated with trisaccharide **4** (1 or 10 nM) or 40 ng/mL of NGF for 5 to 60 min (**A**,**C**); or with 0 to 100 nM of trisaccharide **4** for 5 min (**B**) after pre-incubation with serum-free medium for 1 h. Cell lysates (3 µg of total protein in each lane) were separated by SDS-PAGE. The expression levels of ERK, p-ERK, Akt, and p-Akt were quantified by densitometric analysis of western blot and results were expressed as the ratio of phosphorylated forms (p-ERK or p-Akt) to non-phosphorylated forms (ERK or Akt). Trisaccharide **4** evoked rapid, dose-dependent phosphorylation of ERK 1/2 to an extent similar to that of NGF (**A**,**B**); although **4** showed no effect on Akt phosphorylation (**C**). The graphs are expressed as the mean ± S.D. from five (**A**,**C**) or six (**B**) independent experiments. * *p* < 0.05 with Dunnett’s test compared with the 0 min group (**A**,**C**) or the 0 nM group (**B**).

Many researchers have reported that neuritogenesis of SH-SY5Y is often accompanied by activation of the MAPK/ERK and phosphatidylinositide 3-kinase (PI3K)/Akt signaling cascade after stimulation with growth factors, such as NGF, BDNF, and retinoic acid [8,9,10,11,12], and the signal transductions mediated by MAPK/ERK and PI3K/Akt are thought to be important for cell survival and neuronal differentiation. Therefore, we investigated whether trisaccharide **4** also promotes phosphorylation of ERK 1/2 and Akt. Phosphorylation of ERK 1/2 was rapidly induced by addition of 1 or 10 nM of trisaccharide **4**. The ratio of phosphorylated ERK (p-ERK) to ERK reached a maximum 5 min after stimulation, and then decreased gradually (Figure 4A); however, the ratios varied considerably between experiments when 10 nM of trisaccharide **4** was added (Figure 4A). Although 1 nM of trisaccharide **4** showed a slightly lower value against 40 ng/mL of NGF (157.3 ± 16.9% *vs.* 216.0 ± 15.8%), the increase in the relative ratio of p-ERK to ERK (p-ERK/ERK) at 5 min was statistically significant (*p* < 0.05, Dunnett’s test) compared with 0 min (Figure 4A).

Trisaccharide **4** also induced phosphorylation of ERK after 5 min incubation dose-dependently. The relative ratio of p-ERK/ERK increased in a dose-dependent manner and reached a maximum value at 1 nM (*p* < 0.05, Dunnett’s test), but decreased at higher concentrations (Figure 4B). The dose dependency of ERK phosphorylation corresponded well to the results in Figure 3. However, trisaccharide **4** did not induce Akt phosphorylation significantly, although 40 ng/mL of NGF increased the relative ratio of phosphorylated Akt to Akt (p-Akt/Akt) 5 min after stimulation (547.7 ± 100.7%) (Figure 4C).

#### 2.2.4. U0126 Inhibits Trisaccharide **4**-Promoted ERK Phosphorylation and Neurite Extension

**Figure 5:**
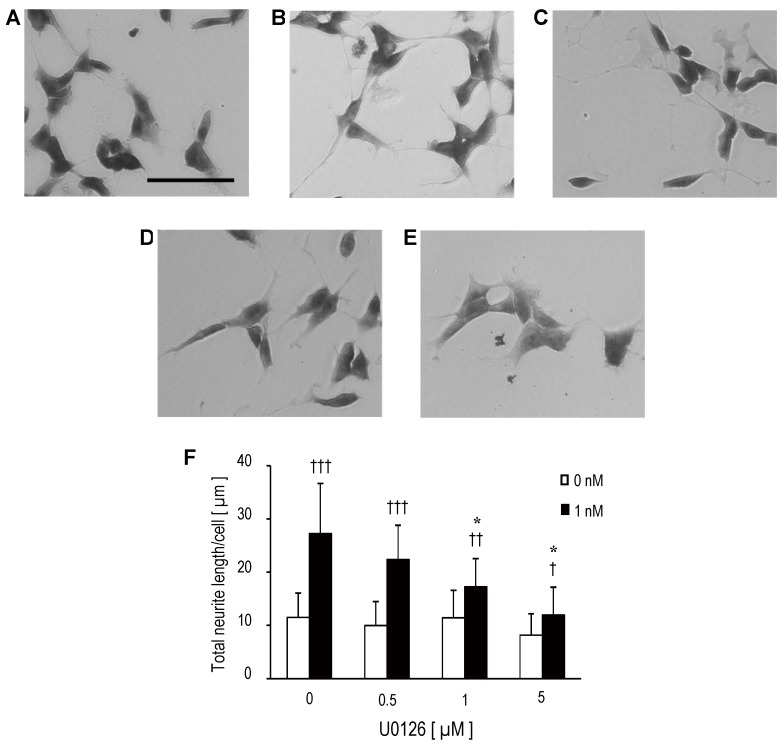
Effects of MEK inhibitor U0126 on neurite extension and ERK 1/2 phosphorylation. SH-SY5Y cells were incubated with trisaccharide **4** (**A**) 0 nM; (**B**–**E**) 1 nM and U0126 (**A**, **B**) 0 µM; (**C**) 0.5 µM; (**D**) 1 µM; (**E**) 5 µM for 3 days in medium containing 1% FBS. Mean total neurite length per cell of 180 cells was measured at each dose (**F**). Statistical significances were determined by Dunnett’s test compared with U0126 0 µM within each trisaccharide **4**-treated group (0 or 1 nM group) (* *p* < 0.05) and by *t*-test (0 *vs.* 1 nM at each U0126 dose level) (^†††^ *p* < 0.001, ^††^ *p* < 0.01, and ^†^ *p* < 0.05).

**Figure 6:**
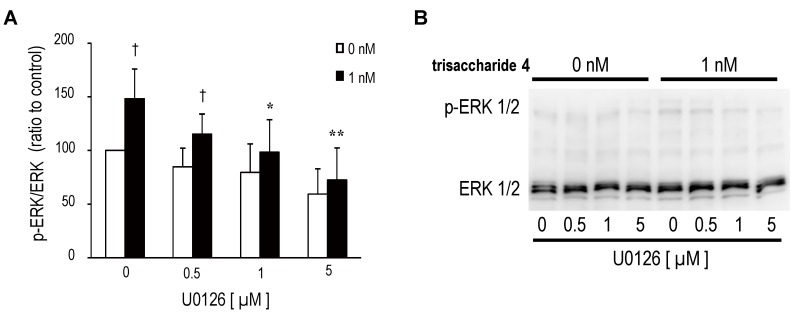
Effects of MEK inhibitor U0126 on neurite extension and ERK 1/2 phosphorylation. (**A**) SH-SY5Y cells were incubated with trisaccharide **4** (0 or 1 nM) for 5 min after pre-incubation with serum-free medium containing U0126 for 1 h, and cell lysates were analyzed as described in Figure 4 (*n* = 5). The error bar represents the standard deviation (S.D.). Statistical significances were determined by Dunnett’s test compared with U0126 0 µM within each trisaccharide **4**-treated group (0 or 1 nM group) (** *p* < 0.01 and * *p* < 0.05) and by *t*-test (0 *vs.* 1 nM at each U0126 dose level) (^†^ *p* < 0.05); (**B**) ERK 1/2 phosphorylation was investigated by Phos-tag SDS-PAGE.

Trisaccharide **4** promoted neurite extension of SH-SY5Y significantly at 1 nM (Figure 5A,B,F), and this effect was abolished by pretreatment with MAPK inhibitor U0126, which inhibits enzymes MEK1 and MEK2 (Figure 5C,D,E). The inhibitory effect of U0126 on trisaccharide **4**-induced neurite extension was dose-dependent (Figure 5F). In addition, U0126 inhibited the phosphorylation of ERK 1/2 in response to trisaccharide **4** dose-dependently (Figure 6A,B), and this response resembled that observed in the neurite extension inhibitory effect (Figure 5F). Therefore, we can infer that trisaccharide **4** stimulates neuritogenesis in SH-SY5Y via activation of the ERK signal cascade, not the PI3K/Akt pathway.

The effect of trisaccharide **4** on neuritogenesis was slightly weaker than that of NGF in PC12 cells (Supplemental Figure 1). This may be accounted for by the fact that trisaccharide **4** activates only the MEK/ERK signaling pathway, unlike NGF, which can activate both MEK/ERK and PI3K/Akt signaling cascades to elongate neurites. Because trisaccharide **4** exerts a prompt effect on SH-SY5Y cells at concentrations as low as 1 nM (Figure 3 and Figure 4A,B), this suggests the presence of specific receptors that recognize trisaccharide **4** on the cell surface of SH-SY5Y. Alternatively, trisaccharide **4** may interact with other receptors specifically or non-specifically and modify their functions. In this scenario, the receptors may be growth factor receptors because fetal bovine serum used in cell culture will inevitably contain growth factors such as PDGF, IGF, EGF, insulin, fibroblast growth factor-2, and transforming growth factor beta 1. Trisaccharide **4** may exert its effect by mimicking, enhancing, or inhibiting these growth factors by interacting with their receptors. There are several reports suggesting the presence of glycoreceptors on the cell surface that recognize the oligosaccharide portion of the ganglioside and elicit biological responses [18,21,22]. Our results strongly support this idea, and we defined the precise sugar structure from which the neuritogenic activity of the LLG-3 ganglioside originated.

## 3. Experimental Section

### 3.1. Synthesis of LLG-3 Glycan Analogues

#### 3.1.1. General Methods

All reactions were carried out under a positive pressure of argon, unless otherwise noted. All chemicals were purchased from Wako Chemicals Inc. (Miyazaki, Japan), Tokyo Chemical Industry Co., Ltd. (Tokyo, Japan), or Sigma-Aldrich Co. (St. Louis, MO, USA) and used without further purification, unless otherwise noted. Molecular sieves were purchased from Wako Chemicals Inc. (Miyazaki, Japan) and pre-dried at 300 °C for 2 h in a muffle furnace, and dried in a flask at 300 °C for 2 h in vacuo prior to use. Dry solvents for reaction media (CH_2_Cl_2_, toluene, THF, CH_3_CN, DMF, pyridine) were purchased from Kanto Chemical Co. Inc. (Tokyo, Japan) and used without purification. Other solvents for reaction media were dried over molecular sieves and used without purification. TLC analysis was performed on Merck TLC plates (silica gel 60F254 on glass plate). Compound detection was either by exposure to UV light (253.6 nm) or by soaking in a solution of 10% H_2_SO_4_ in ethanol followed by heating. Silica gel (80 mesh and 300 mesh; Fuji Silysia Co. (Aichi, Japan) ) was used for flash column chromatography. The quantity of silica gel was usually 100 to 200 times the weight of the crude sample. Solvent systems for chromatography were specified as *v*/*v* ratios. Evaporation and concentration were carried out *in vacuo*. ^1^H-NMR and ^13^C-NMR spectra were recorded on 400 MHz (JEOL ECX400), 500 MHz (Biospin AVANCE III, Bruker, Billerica, MA, USA) or 600 MHz (JEOL ECA600) spectrometers. Chemical shifts in ^1^H-NMR spectra are expressed in ppm (δ) relative to the Me_4_Si signal, adjusted to δ 0.00 ppm. Data are presented as follows: chemical shift, multiplicity (s = singlet, d = doublet, t = triplet, q = quartet, dd = double doublet, td = triple doublet, m = multiplet and/or multiple resonances), integration, coupling constant in hertz (Hz), and position of the corresponding proton. COSY methods were used to confirm the NMR peak assignments. High-resolution mass (ESI-TOF MS) spectra were obtained with a mass spectrometer (micrOTOF, Bruker, Billerica, MA, USA). Optical rotations were measured with a high-sensitivity polarimeter (SEPA-300, Horiba, Kyoto, Japan).

#### 3.1.2. Experimental Procedures

**2-(Trimethylsilyl)ethyl [methyl 4,7,8,9-tetra-*O*-acetyl-3,5-dideoxy-5-(methyl 4,7,9-tri-*O*-acetyl-5-diacetamido-3,5-dideoxy-8-*O*-methyl-d-*glycero*-α-d-*galacto*-2-nonulopyranosylonate)oxyacetamido-d-*glycero*-α-d-*galacto*-2-nonulopyranosylonate]-(2→3)-(4-*O*-acetyl-2,6-di-*O*-benzoyl-β-d-galactopyranosyl)-(1→4)-2,3,6-tri-*O*-benzyl-β-d-glucopyranoside (9).** To a mixture of **7** (41.2 mg, 26.6 μmol) and **8** (29.2 mg, 53.2 μmol) in CH_2_Cl_2_ (1.0 mL) were added 4 Å molecular sieves (AW300, 60 mg) at room temperature. After stirring for 30 min, the mixture was cooled to –40 °C. TMSOTf (0.1 µL, 5.32 µmol) was then added to the mixture at −40 °C. After stirring for 5 min at −40 °C as the reaction was monitored by TLC (20:1 CHCl_3_–MeOH), the reaction was quenched by the addition of triethylamine. The solution was diluted with CHCl_3_ and filtered through Celite. The filtrate was then washed with satd. aq. NaHCO_3_ and brine. The organic layer was subsequently dried over Na_2_SO_4_, and concentrated. The residue was purified by silica gel column chromatography (50:1 to 40:1 toluene–MeOH) to give **9** (41.8 mg, 81%): [α]_D_ +10.0° (c 0.9, CHCl_3_); ^1^H NMR (600 MHz, CDCl_3_) δ 8.25–7.17 (m, 25H, 5Ar), 6.03 (d, 1H, *J*_NH,5_ = 10.3 Hz, NH^*b*^), 5.69 (m, 1H, H-8^*b*^), 5.58 (td, 1H, *J*_3eq,4_ = 5.2 Hz, *J*_3ax,4_ = *J*_4,5_ = 10.0 Hz, H-4^*a*^), 5.31 (dd, 1H, *J*_1,2_ = 8.0 Hz, *J*_2,3_ = 10.3 Hz, H-2^*c*^), 5.20 (d, 1H, H-1^*c*^), 5.11 (dd, 1H, *J*_6,7_ = 2.8 Hz, *J*_7,8_ = 9.8 Hz, H-7^*b*^), 5.07 (d, 1H, *J*_3,4_ = 3.5 Hz, H-4^*c*^), 4.98 (dd, 1H, *J*_6,7_ = 1.7 Hz, *J*_5,6_ = 10.3 Hz, H-6^*a*^), 4.93 (dd, 1H, *J*_7,8_ = 8.0 Hz, H-7^*a*^), 5.02 and 4.89 (2d, 2H, PhC*H_2_*), 4.87 and 4.66 (2d, 2H, PhC*H*_2_), 4.83 (m, 1H, H-4^*b*^), 4.76 (dd, 1H, H-3^*c*^), 4.39–4.21 (m, 6H, PhC*H_2_*, H-9a^*b*^, H-6^*b*^, H-4^*d*^, H-5^*a*^), 4.12–3.85 (m, 11H, OCH_2_, H-9a^*a*^, H-9b^*a*^, OC*H*_2_CH_2_Si, H-1^*d*^, H-3^*d*^, H-5^*b*^, CO_2_Me), 3.76 (s, 3H, CO_2_Me), 3.70–3.59 (m, 2H, H-2^*d*^, H-6a^*c*^), 3.54 (m, 1H, OC*H*_2_CH_2_Si), 3.47–3.33 (m, 6H, H-6b^*d*^, H-5^*d*^, H-6b^*c*^, OMe), 2.88 (dd, 1H, *J*_3eq,4_ = 5.1 Hz, *J*_gem_ = 13.2 Hz, H-3eq^*a*^), 2.59 (dd, 1H, *J*_3eq,4_ = 3.6 Hz, *J*_gem_ = 12.1 Hz, H-3eq^*b*^), 2.38–1.96 (m, 30H, 10Ac), 1.93 (dd, 1H, *J*_3ax,4_ = 10.3 Hz, H-3ax^*a*^), 1.71 (t, 1H, *J*_3ax,4_ = 12.1 Hz, H-3ax^*b*^), 1.02–0.98 (m, 2H, OCH_2_C*H_2_*Si), 0.00 (s, 9H, SiMe_3_); ^13^C NMR (150 MHz, CDCl_3_) δ 176.5, 175.8, 173.0, 172.9, 172.6, 172.5, 172.4, 172.3, 172.2, 172.1, 171.1, 170.3, 169.8, 167.6, 167.3, 141.4, 140.9, 140.8, 135.4, 135.2, 132.6, 132.3, 132.0, 131.9, 131.9, 130.8, 130.5, 130.4, 130.4, 130.4, 130.4, 130.4, 130.3, 130.3, 130.2, 130.2, 129.7, 129.5, 129.4, 129.4, 129.4, 129.3, 129.3, 129.3, 129.2, 105.1, 103.0, 100.9, 99.1, 85.2, 84.2, 76.3, 74.6, 74.3, 74.0, 72.5, 71.8, 71.5, 71.3, 70.2, 70.2, 69.0, 68.8, 67.7, 67.1, 67.1, 66.8, 66.7, 66.3, 63.2, 61.4, 61.2, 60.9, 57.8, 57.8, 56.3, 52.9, 52.8, 47.8, 37.8, 37.2, 29.4, 27.7, 25.8, 21.1, 20.6, 20.5, 20.5, 20.5, 20.4, 20.1, 18.2, 0.0, 0.0, 0.0; HRMS (ESI) *m/z*: found [M + Na]^+^ 1961.6972, C_95_H_118_N_2_O_39_Si calcd for [M + Na]^+^ 1961.6973.

**2-(Trimethylsilyl)ethyl [5-(5-acetamido-3,5-dideoxy-8-*O*-methyl-d-*glycero*-α-d-*galacto*-2-nonulopyranosylonic acid)oxyacetamido-3,5-dideoxy-d-*glycero*-α-d-*galacto*-2-nonulopyranosylonic acid]-(2→3)-(β-d-galactopyranosyl)-(1→4)-β-d-glucopyranoside (2)**. To a solution of **9** (39.6 mg, 21.0 µmol) in EtOAc (1.0 mL) was added Pd(OH)_2_/C (20%, 40.0 mg) at room temperature. After stirring for 2 h at room temperature under a hydrogen atmosphere as the reaction was monitored by TLC (20:1 CHCl_3_–MeOH), the mixture was filtered through Celite. The filtrate was concentrated, and the crude residue was exposed to high vacuum. The resulting residue was then dissolved in pyridine (1.0 mL), and lithium chloride (5.2 mg, 126 µmol) was added at room temperature. After stirring for 18 h under reflux as the reaction was monitored by TLC (5:2:0.2 CHCl_3_–MeOH–H_2_O), the reaction mixture was co-evaporated with toluene. The crude residue was purified by gel filtration column chromatography (Sephadex LH-20) using MeOH as eluent. The purified product was exposed to high vacuum, and then dissolved in 0.1 M aq NaOH (2.1 mL). After stirring for 42 h at room temperature and another 10 h at 40 °C as the reaction was monitored by TLC (4:6:1 CHCl_3_–MeOH–H_2_O), the reaction mixture was neutralized with Dowex (H^+^) resin. The resin was filtered through cotton wool and the filtrate was then evaporated. The residue was purified by silica gel column chromatography (12:8:1 to 4:6:1 CHCl_3_–MeOH–H_2_O), followed by gel filtration column chromatography (Sephadex LH-20) using MeOH–H_2_O (5:1) as eluent to give **2** (10.7 mg, 49%): [α]_D_ –41.9° (c 0.2, MeOH); ^1^H NMR (500 MHz, CD_3_OD) δ 4.45 (d, 1H, *J*_1,2_ = 7.6 Hz, H-1), 4.33 (d, 1H, *J*_1,2_ = 8.2 Hz, H-1), 4.34 and 4.27 (2d, 2H, OCH_2_), 4.04–3.21 (m, 31H, H-4^*a*^, H-5^*a*^, H-6^*a*^, H-7^*a*^, H-8^*a*^, H-9a^*a*^, H-9b^*a*^, H-4^*b*^, H-5^*b*^, H-6^*b*^, H-7^*b*^, H-8^*b*^, H-9a^*b*^, H-9b^*b*^, H-2^*c*^, H-3^*c*^, H-4^*c*^, H-5^*c*^, H-6a^*c*^, H-6b^*c*^, H-2^*d*^, H-3^*d*^, H-4^*d*^, H-5^*d*^, H-6a^*d*^, H-6b^*d*^, OC*H_2_*CH_2_Si, OMe), 2.78 (dd, 1H, *J*_3eq,4_ = 3.4 Hz, *J*_gem_ = 12.6 Hz, H-3eq), 2.49 (dd, 1H, *J*_3eq,4_ = 4.6 Hz, *J*_gem_ = 12.6 Hz, H-3eq), 2.00 (s, 3H, Ac), 1.77–1.69 (m, 2H, H-3ax^*a*^, H-3ax^*b*^), 1.07–0.92 (m, 2H, OCH_2_C*H_2_*Si), 0.00 (s, 9H, SiMe_3_); ^13^C NMR (125 MHz, CD_3_OD) δ 175.5, 174.8, 174.8, 174.5, 104.5, 103.3, 101.5, 101.1, 81.4, 80.3, 77.2, 76.7, 76.2, 74.4, 74.3, 74.0, 73.0, 71.1, 70.8, 69.3, 69.0, 69.0, 69.0, 68.8, 68.6, 63.8, 63.7, 62.5, 61.6, 61.4, 59.1, 53.7, 53.5, 41.4, 40.3, 22.9, 19.0, −1.4, −1.4, −1.4; HRMS (ESI) *m/z*: found [M − 2H + Na]^−^ 1075.3631, C_40_H_70_N_2_O_28_Si calcd for [M − 2H + Na]^−^ 1075.3631.

**4-Methoxyphenyl [methyl 4,7,8,9-tetra-*O*-acetyl-3,5-dideoxy-5-(methyl 5-acetamido-4,7,8,9-tetra-*O*-acetyl-3,5-dideoxy-d-*glycero*-α-d-*galacto*-2-nonulopyranosylonate)oxyacetamido-d-*glycero*-α-d-*galacto*-2-nonulopyranosylonate]-(2→3)-2,6-di-*O*-benzyl-β-d-galactopyranoside (12).** To a solution of **10** (414 mg, 735 μmol) and **11** (788 mg, 878 μmol) in acetonitrile (15.1 mL) were added EDC·HCl (260 mg, 1.36 mmol) and HOBt (50.8 mg, 377 μmol) at room temperature. After stirring for 7 h at room temperature as the reaction was monitored by TLC (15:1 CHCl_3_–MeOH), the mixture was diluted with EtOAc. The solution was then washed with 2 M HCl, satd. aq. NaHCO_3_ and brine. The organic layer was subsequently dried over Na_2_SO_4_, and concentrated. The resulting residue was purified by silica gel column chromatography (55:1 CHCl_3_–MeOH) to give **12** (762 mg, 71%): [α]_D_ +3.5° (c 1.0, CHCl_3_); ^1^H NMR (500 MHz, CDCl_3_) δ 7.42–6.78 (m, 14H, 3Ar), 6.10 (d, 1H, *J*_NH,5_ = 10.0 Hz, NH^*b*^), 5.43 (td, 1H, *J*_8,9a_ = 3.0 Hz, *J*_7,8_ = *J*_8,9b_ = 10.5 Hz, H-8^*a*^), 5.33–5.32 (m, 2H, H-7^*b*^, H-8^*b*^), 5.23 (dd, 1H, *J*_6,7_ = 2.3 Hz, H-7^*a*^), 5.04 (d, 1H, *J*_NH,5_ = 10.0 Hz, NH^*a*^), 4.95–4.87 (m, 4H, H-4^*a*^, H-4^*b*^, H-1^*c*^, PhC*H*_2_), 4.82 (d, 1H, *J*_gem_ = 11.5 Hz, PhC*H*_2_), 4.57 (2d, 2H, *J*_gem_ = 11.5 Hz, PhC*H_2_*), 4.34 (dd, 1H, *J*_gem_ = 12.5 Hz, H-9a^*a*^), 4.29–4.26 (m, 2H, H-9a^*b*^, H-3^*c*^), 4.18–4.06 (m, 6H, H-5^*a*^, H-6^*a*^, H-5^*b*^, H-6^*b*^, H-9b^*b*^, OCH_2_), 3.95 (dd, 1H, H-9b^*a*^), 3.86–3.76 (m, 15H, H-2^*c*^, H-4^*c*^, H-5^*c*^, H-6a^*c*^, H-6b^*c*^, OCH_2_, 3OMe), 2.72 (d, 1H, *J*_OH,4_ = 3.0 Hz, OH^*c*^), 2.67 (dd, 1H, *J*_3eq,4_ = 5.0 Hz, *J*_gem_ = 13.0 Hz, H-3eq^*a*^), 2.62 (dd, 1H, *J*_3eq,4_ = 4.5 Hz, *J*_gem_ = 12.5 Hz, H-3eq^*b*^), 2.14–1.89 (m, 29H, H-3ax^*a*^, H-3ax^*b*^, 9Ac); ^13^C NMR (125 MHz, CDCl_3_) δ 170.9, 170.6, 170.5, 170.3, 170.2, 170.0, 170.0, 169.8, 168.6, 167.6, 155.2, 151.7, 138.9, 138.2, 128.3, 128.2, 127.9, 127.6, 127.6, 127.4, 118.6, 114.5, 102.8, 98.5, 98.1, 75.7, 75.0, 73.6, 73.1, 73.0, 72.9, 69.4, 68.8, 68.5, 68.2, 67.5, 67.2, 63.8, 62.6, 62.2, 55.6, 53.2, 53.1, 49.3, 48.6, 37.5, 37.0, 23.2, 21.2, 21.0, 20.8, 20.8, 20.7, 20.7; HRMS (ESI) *m/z*: found [M + Na]^+^ 1451.4899, C_67_H_84_N_2_O_32_ calcd for [M + Na]^+^ 1451.4899.

**4-Methoxyphenyl [methyl 4,7,8,9-tetra-*O*-acetyl-3,5-dideoxy-5-(methyl 5-acetamido-4,7,8,9-tetra-*O*-acetyl-3,5-dideoxy-d-*glycero*-α-d-*galacto*-2-nonulopyranosylonate)oxyacetamido-d-*glycero*-α-d-*galacto*-2-nonulopyranosylonate]-(2→3)-2,4,6-tri-*O*-benzoyl-β-d-galactopyranoside (13).** To a solution of **12** (650 mg, 455 µmol) in EtOAc (9.1 mL) was added Pd(OH)_2_/C (20%, 650 mg) at room temperature. After stirring for 30 min at room temperature under a hydrogen atmosphere as the reaction was monitored by TLC (15:1 CHCl_3_–MeOH), the mixture was filtered through Celite. The filtrate was concentrated, and the crude residue was exposed to high vacuum. Then, it was dissolved in pyridine (2.3 mL). Benzoic anhydride (618 mg, 2.73 mmol) and DMAP (5.6 mg, 45.5 µmol) were added to the mixture at 0 °C. After stirring for 11 h at room temperature as the reaction was monitored by TLC (15:1 CHCl_3_–MeOH), the reaction was quenched by the addition of MeOH at 0 °C. The mixture was co-evaporated with toluene and the residue was then diluted with CHCl_3_, and washed with 2 M HCl, H_2_O and satd. aq. NaHCO_3_. The organic layer was subsequently dried over Na_2_SO_4_, and concentrated. The resulting residue was purified by silica gel column chromatography (50:1 CHCl_3_–MeOH) to give 13 (666 mg, 94%): [α]_D_ +48.0° (c 1.0, CHCl_3_); ^1^H NMR (500 MHz, CDCl_3_) δ 8.18–6.68 (m, 19H, 4Ar), 5.94 (d, 1H, *J*_NH,5_ = 10.4 Hz, NH^*b*^), 5.69 (dd, 1H, *J*_1,2_ = 8.0 Hz, *J*_2,3_ = 10.1 Hz, H-2^*c*^), 5.63 (td, 1H, *J*_8,9a_ = 2.5 Hz, *J*_8,9b_ = 6.4 Hz, *J*_7,8_ = 9.6 Hz, H-8^*b*^), 5.40 (d, 1H, *J*_3,4_ = 3.3 Hz, H-4^*c*^), 5.34–5.26 (m, 3H, H-7^*a*^, H-8^*a*^, H-1^*c*^), 5.14–5.10 (m, 2H, NH^*a*^, H-7^*b*^), 4.97 (dd, 1H, H-3^*c*^), 4.90 (td, 1H, *J*_3eq,4_ = 4.7 Hz, *J*_3ax,4_ = *J*_4,5_ = 10.3 Hz, H-4^*a*^), 4.84 (td, 1H, *J*_3eq,4_ = 4.5 Hz, *J*_3ax,4_ = *J*_4,5_ = 10.6 Hz, H-4^*b*^), 4.49 (dd, 1H, *J*_5,6a_ = 7.6 Hz, *J*_gem_ = 11.4 Hz, H-6a^*c*^), 4.43 (dd, 1H, *J*_5,6b_ = 5.6 Hz, H-6b^*c*^), 4.31–4.23 (m, 3H, H-5^*c*^, H-9a^*a*^, H-9a^*b*^), 4.13 (dd, 1H, *J*_6,7_ = 2.0 Hz, *J*_5,6_ = 10.8 Hz, H-6^*a*^), 4.08–4.02 (m, 3H, H-5^*a*^, H-9b^*a*^, OCH_2_), 3.93–3.86 (m, 5H, H-5^*b*^, H-9b^*b*^, OMe), 3.82 (s, 3H, OMe), 3.75–3.69 (m, 5H, H-6^*b*^, OCH_2_, OMe), 2.64 (dd, 1H, *J*_gem_ = 12.9 Hz, H-3eq^*a*^), 2.55 (dd, 1H, *J*_gem_ = 12.7 Hz, H-3eq^*b*^), 2.24–1.88 (m, 25H, 8Ac, H-3ax^*a*^), 1.68 (t, 1H, H-3ax^*b*^), 1.44 (s, 3H, Ac); ^13^C NMR (125 MHz, CDCl_3_) δ 170.9, 170.6, 170.6, 170.3, 170.2, 170.1, 170.0, 169.8, 168.5, 168.3, 167.6, 165.9, 165.4, 155.5, 151.5, 133.4, 133.2, 133.1, 130.2, 130.1, 129.8, 129.3, 128.5, 128.5, 128.4, 119.0, 114.4, 101.2, 98.4, 96.9, 77.6, 72.9, 71.9, 71.3, 71.0, 68.9, 68.8, 68.5, 68.4, 67.5, 67.1, 66.7, 63.8, 68.5, 68.4, 67.5, 67.1, 66.7, 63.8, 62.7, 62.5, 62.2, 55.6, 53.3, 53.2, 49.3, 48.2, 37.5, 37.4, 23.2, 21.5, 21.0, 20.8, 20.7, 20.6, 20.3; HRMS (ESI) *m/z*: found [M + Na]^+^ 1583.4748, C_74_H_84_N_2_O_35_ calcd for [M + Na]^+^ 1583.4747.

**[Methyl 4,7,8,9-tetra-*O*-acetyl-3,5-dideoxy-5-(methyl 5-acetamido-4,7,8,9-tetra-*O*-acetyl-3,5-dideoxy-d-*glycero*-α-d-*galacto*-2-nonulopyranosylonate)oxyacetamido-d-*glycero*-α-d-*galacto*-2-nonulopyranosylonate]-(2→3)-2,4,6-tri-*O*-benzoyl-d-galactopyranose (14).** To a suspension of **13** (200 mg, 128 µmol) in acetonitrile/toluene/H_2_O (6:5:3, 2.6 mL) was added CAN (702 mg, 1.28 mmol) at 0 °C. After stirring for 5 min at 0 °C as the reaction was monitored by TLC (30:1 CHCl_3_–MeOH), the mixture was diluted with EtOAc, and then washed with H_2_O, satd. aq. NaHCO_3_ and brine. The organic layer was subsequently dried over Na_2_SO_4_, and concentrated. The residue was roughly purified by silica gel column chromatography (45:1 CHCl_3_–MeOH). The crude product was exposed to high vacuum, and then dissolved in pyridine (1.0 mL). Benzoic anhydride (66.3 mg, 293 µmol) and DMAP (1.2 mg, 9.8 µmol) were added to the solution at 0 °C. After stirring for 5.5 h at room temperature as the reaction was monitored by TLC (30:1 CHCl_3_–MeOH, developed twice), the reaction was quenched by the addition of MeOH at 0 °C. The mixture was co-evaporated with toluene, and the residue was then diluted with CHCl_3_, and washed with 2 M HCl, H_2_O and satd. aq. NaHCO_3_. The organic layer was subsequently dried over Na_2_SO_4_, and concentrated. The resulting residue was purified by silica gel column chromatography (50:1 CHCl_3_–MeOH). The obtained product was exposed to high vacuum, and then dissolved in DMF (890 µL). Hydrazine acetate (1.2 mg, 9.8 µmol) was added to the solution at 0 °C. After stirring for 9 h at room temperature as the reaction was monitored by TLC (30:1 CHCl_3_–MeOH, developed twice), the reaction mixture was diluted with EtOAc, and washed with H_2_O and brine. The organic layer was subsequently dried over Na_2_SO_4_, and concentrated. The resulting residue was purified by silica gel column chromatography (40:1 CHCl_3_–MeOH) to give 14 (96.2 mg, 52%, anomeric mixture): ^1^H NMR (500 MHz, CDCl_3_) δ 8.23–7.39 (m, 30H, 6Ph), 6.03 (d, 2H, *J*_NH,5_ = 10.3 Hz, NH^*b*^, NH^*b*^), 5.68–5.63 (m, 2H, H-8^*b*^, H-8^*b*^), 5.60–5.58 (m, 2H, H-2^*c*^, H-2^*c*^), 5.50 (d, 1H, *J*_3,4_ = 2.8 Hz, H-4^*c*^), 5.46 (d, 1H, *J*_3,4_ = 3.0 Hz, H-4^*c*^), 5.34–5.19 (m, 9H, H-8^*a*^, H-8^*a*^, H-7^*a*^, H-7^*a*^, NH^*a*^, NH^*a*^, H-1^*c*^, H-7^*b*^, H-7^*b*^), 5.14–5.08 (m, 2H, H-1^*c*^, H-3^*c*^), 5.03 (dd, 1H, *J*_2,3_ = 10.3 Hz, H-3^*c*^), 4.93–4.86 (m, 4H, H-4^*a*^, H-4^*a*^, H-4^*b*^, H-4^*b*^), 4.61 (m, 1H, H-5^*c*^), 4.53–4.45 (m, 3H, H-6a^*c*^, H-6a^*c*^, H-5^*c*^), 4.36–4.25 (m, 6H, H-6b^*c*^, H-9a^*b*^, H-9a^*b*^, H-6^*c*^, H-9a^*a*^, H-9a^*a*^), 4.15–3.96 (m, 12H, H-9b^*a*^, 2OC*H*_2_, H-5^*a*^, H-5^*a*^, H-5^*b*^, H-5^*b*^, H-9b^*a*^, H-6^*a*^, H-6^*a*^, H-9b^*b*^, H-9b^*b*^), 3.89, 3.84 and 3.83 (3s, 12H, 4OMe), 3.79–3.74 (m, 4H, 2OC*H*_2_, H-6^*b*^, H-6^*b*^), 2.67–2.63 (m, 2H, H-3eq^*a*^, H-3eq^*a*^), 2.55–2.51 (m, 2H, H-3eq^*b*^, H-3eq^*b*^), 2.22–1.88 (m, 50H, 16Ac, H-3ax^*a*^, H-3ax^*a*^), 1.70–1.66 (m, 7H, 2Ac, H-3ax^*b*^), 1.65 (t, 1H, *J*_3ax,4_ = *J*_gem_ = 12.4 Hz, H-3ax^*b*^); ^13^C NMR (125 MHz, CDCl_3_) δ 171.4, 171.0, 170.9, 170.7, 170.6, 170.3, 170.3, 170.2, 170.2, 170.0, 169.9, 168.7, 168.7, 168.3, 168.2, 167.6, 167.3, 165.9, 165.9, 165.9, 165.7, 133.5, 133.4, 133.3, 133.1, 133.1, 130.3, 130.2, 130.0, 129.9, 129.8, 129.8, 129.5, 129.3, 128.6, 128.6, 128.5, 128.3, 128.3, 98.4, 97.3, 97.0, 96.1, 92.0, 77.6, 73.5, 72.9, 72.5, 72.3, 71.2, 70.8, 69.9, 69.5, 68.8, 68.6, 68.5, 68.1, 67.7, 67.1, 67.0, 66.9, 63.7, 62.7, 62.6, 62.3, 62.2, 53.3, 53.3, 53.2, 49.3, 48.4, 37.7, 37.6, 37.4, 31.9, 29.7, 29.4, 23.2, 22.7, 21.5, 21.4, 21.0, 20.8, 20.7, 20.6; HRMS (ESI) *m/z*: found [M + Na]^+^ 1477.4329, C_67_H_78_N_2_O_34_ calcd for [M + Na]^+^ 1477.4328.

**[Methyl 4,7,8,9-tetra-*O*-acetyl-3,5-dideoxy-5-(methyl 5-acetamido-4,7,8,9-tetra-*O*-acetyl-3,5-dideoxy-d-*glycero*-α-d-*galacto*-2-nonulopyranosylonate)oxyacetamido-d-*glycero*-α-d-*galacto*-2-nonulopyranosylonate]-(2→3)-2,4,6-tri-*O*-benzoyl-d-galactopyranosyl trichloroacetimidate (15)**. To a solution of **14** (96.2 mg, 66.1 µmol) in CH_2_Cl_2_ (1.3 mL) were added trichloroacetonitrile (132 µL, 1.32 mmol) and DBU (2.0 µL, 13.2 µmol) at 0 °C. After stirring for 1.5 h at 0 °C as the reaction was monitored by TLC (30:1 CHCl_3_–MeOH, developed twice), the reaction mixture was evaporated. The resulting residue was purified by silica gel column chromatography (50:1 CHCl_3_–MeOH) to give **15** (85.8 mg, 81%, α:β = 1:1.4): ^1^H NMR (500 MHz, CDCl_3_); α isomer: δ 8.62 (s, 1H, C=NH), 8.19–7.36 (m, 15H, 3Ph), 6.87 (d, 1H, *J*_1,2_ = 3.8 Hz, H-1^*c*^), 6.10 (d, 1H, *J*_NH,5_ = 10.4 Hz, NH^*b*^), 5.72 (d, 1H, *J*_3,4_ = 3.0 Hz, H-4^*c*^), 5.67–5.62 (m, 2H, H-8^*b*^, H-2^*c*^), 5.52 (dd, 1H, *J*_2,3_ = 10.6 Hz, H-3^*c*^), 5.35 (dd, 1H, *J*_6,7_ = 2.2 Hz, H-7^*b*^), 5.32–5.25 (m, 3H, H-7^*a*^, H-8^*a*^, NH^*a*^), 4.92–4.85 (m, 2H, H-4^*a*^, H-4^*b*^), 4.52 (dd, 1H, *J*_5,6a_ = 6.4 Hz, *J*_gem_ = 11.2 Hz, H-6a^*c*^), 4.35 (m, 1H, H-6b^*c*^), 4.26–4.21 (m, 2H, H-5^*c*^, H-9a^*b*^), 4.19–4.06 (m, 6H, H-5^*b*^, H-9b^*b*^, H-9a^*a*^, OCH_2_, H-5^*a*^, H-9b^*a*^), 3.96 (dd, 1H, *J*_6,7_ = 2.2 Hz, *J*_5,6_ = 10.7 Hz, H-6^*a*^), 3.86 (s, 3H, OMe), 3.83–3.80 (m, 5H, OCH_2_, H-6^*b*^, OMe), 2.68 (dd, 1H, *J*_3eq,4_ = 4.7 Hz, *J*_gem_ = 12.3 Hz, H-3eq^*a*^), 2.55 (dd, 1H, *J*_3eq,4_ = 4.6 Hz, *J*_gem_ = 12.4 Hz, H-3eq^*b*^), 2.21–1.88 (m, 28H, 9Ac, H-3ax^*a*^), 1.68 (t, 1H, *J*_3ax,4_ = 12.4 Hz, H-3ax^*b*^); β isomer: δ 8.70 (s, 1H, C=NH), 8.19–7.36 (m, 15H, 3Ph), 6.28 (d, 1H, *J*_1,2_ = 8.2 Hz, H-1^*c*^), 5.96 (d, 1H, *J*_NH,5_ = 10.3 Hz, NH^*b*^), 5.72 (m, 1H, H-2^*c*^), 5.64 (m, 1H, H-8^*b*^), 5.48 (d, 1H, *J*_3,4_ = 3.0 Hz, H-4^*c*^), 5.32–5.25 (m, 3H, H-8^*a*^, H-7^*a*^, NH^*a*^), 5.13 (dd, 1H, *J*_6,7_ = 2.6 Hz, *J*_7,8_ = 9.3 Hz, H-7^*b*^), 5.05 (dd, 1H, *J*_2,3_ = 10.0 Hz, H-3^*c*^), 4.93–4.82 (m, 2H, H-4^*a*^, H-4^*b*^), 4.56 (dd, 1H, *J*_5,6a_ = 6.0 Hz, *J*_gem_ = 10.8 Hz, H-6a^*c*^), 4.42 (m, 1H, H-5^*c*^), 4.37–4.33 (m, 2H, H-6b^*c*^, H-9a^*b*^), 4.27 (m, 1H, H-9a^*a*^), 4.09–4.03 (m, 4H, H-6^*a*^, H-9b^*a*^, H-5^*a*^, OCH_2_), 3.94–3.88 (m, 2H, H-9b^*b*^, H-5^*b*^), 3.87 and 3.82 (2s, 6H, 2OMe), 3.74–3.70 (m, 2H, OCH_2_, H-6^*b*^), 2.64 (dd, 1H, *J*_3eq,4_ = 4.6 Hz, *J*_gem_ = 12.7 Hz, H-3eq^*a*^), 2.52 (dd, 1H, *J*_3eq,4_ = 4.4 Hz, *J*_gem_ = 12.3 Hz, H-3eq^*b*^), 2.21–1.88 (m, 28H, 9Ac, H-3ax^*a*^), 1.61 (t, 1H, *J*_3ax,4_ = 12.3 Hz, H-3ax^*b*^); ^13^C NMR (125 MHz, CDCl_3_); anomeric mixture (α:β = 1:1.4) δ 170.8, 170.8, 170.6, 170.6, 170.5, 170.5, 170.3, 170.2, 170.1, 169.9, 169.8, 169.7, 169.6, 168.6, 168.5, 168.2, 167.6, 167.5, 165.8, 165.8, 165.8, 165.7, 165.5, 165.0, 161.1, 160.8, 133.6, 133.3, 133.1, 133.0, 132.9, 130.1, 130.0, 129.9, 129.9, 129.8, 129.7, 129.3, 129.3, 128.6, 128.5, 128.3, 128.2, 128.1, 98.4, 96.9, 96.6, 96.5, 94.2, 90.9, 90.4, 77.2, 72.9, 72.8, 72.0, 71.9, 71.2, 70.1, 69.9, 69.3, 68.8, 68.7, 68.5, 68.1, 67.6, 67.4, 67.3, 67.1, 66.7, 63.7, 63.7, 62.6, 62.5, 62.1, 61.9, 53.2, 53.2, 49.2, 48.7, 48.1, 38.5, 37.4, 37.4, 37.3, 31.9, 29.6, 29.3, 23.1, 22.6, 21.4, 21.2, 21.0, 20.9, 20.8, 20.7, 20.7, 20.6, 20.6, 20.2; HRMS (ESI) *m/z*: found [M + Na]^+^ 1620.3426, C_69_H_78_Cl_3_N_3_O_34_ calcd for [M + Na]^+^ 1620.3425.

**2-(Trimethylsilyl)ethyl [methyl 4,7,8,9-tetra-*O*-acetyl-3,5-dideoxy-5-(methyl 5-acetamido-4,7,8,9-tetra-*O*-acetyl-3,5-dideoxy-d-*glycero*-α-d-*galacto*-2-nonulopyranosylonate)oxyacetamido-d-*glycero*-α-d-*galacto*-2-nonulopyranosylonate]-(2→3)-(2,4,6-tri-*O*-benzoyl-β-d-galactopyranosyl)-(1→4)-2,3,6-tri-*O*-benzyl-β-d-glucopyranoside (16).** To a mixture of **15** (63.3 mg, 39.0 µmol) and **8** (43.6 mg, 79.0 µmol) in CH_2_Cl_2_ (1.6 mL) was added 4 Å molecular sieves (AW300, 150 mg) at room temperature. After stirring for 1 h, the mixture was cooled to 0 °C, and TMSOTf (0.7 µL, 4.0 µmol) was added at 0 °C. After stirring for 2 h at 0 °C as the reaction was monitored by TLC (30:1 CHCl_3_–MeOH), the reaction was quenched by the addition of satd. aq. NaHCO_3_. The solution was diluted with CHCl_3_ and filtered through Celite. The filtrate was then washed with satd. aq. NaHCO_3_ and brine. The organic layer was subsequently dried over Na_2_SO_4_, and concentrated. The resulting residue was purified by silica gel column chromatography (60:1 CHCl_3_–MeOH) to give 16 (55.8 mg, 78%): [α]_D_ +2.4° (c 1.0, CHCl_3_); ^1^H NMR (500 MHz, CDCl_3_) δ 8.25–7.07 (m, 30H, 6Ph), 5.92 (d, 1H, *J*_NH,5_ = 10.4 Hz, NH^*b*^), 5.72 (td, 1H, *J*_8,9a_ = 2.4 Hz, *J*_7,8_ = *J*_8,9b_ = 9.7 Hz, H-8^*b*^), 5.48 (dd, 1H, *J*_1,2_ = 8.0 Hz, *J*_2,3_ = 9.9 Hz, H-2^*c*^), 5.34–5.27 (m, 3H, H-7^*a*^, PhC*H*_2_, H-8^*a*^), 5.23 (d, 1H, H-1^*c*^), 5.15–5.06 (m, 3H, NH^*a*^, H-4^*c*^, H-7^*b*^), 4.93–4.79 (m, 5H, H-4^*a*^, H-3^*c*^, 2PhC*H*_2_, H-4^*b*^), 4.66 (d, 1H, *J*_gem_ = 11.2 Hz, PhC*H*_2_), 4.42 (2d, 2H, *J*_gem_ = 12.0 Hz, PhC*H*_2_), 4.33–4.24 (m, 3H, H-6a^*c*^, H-1^*d*^, H-6^*a*^), 4.15–3.84 (m, 17H, H-9a^*a*^, H-5^*a*^, H-9b^*a*^, H-6a^*d*^, OC*H*_2_CH_2_Si, H-6b^*c*^, H-5^*c*^, H-5^*b*^, OC*H*_2_, H-9a^*b*^, H-9b^*b*^, 2OMe), 3.74–3.61 (m, 4H, H-4^*d*^, H-6^*b*^, H-6b^*d*^, OCH_2_), 3.56–3.51 (m, 2H, OC*H*_2_CH_2_Si, H-3^*d*^), 3.38–3.33 (m, 2H, H-2^*d*^, H-5^*d*^), 2.65 (dd, 1H, *J*_3eq,4_ = 4.7 Hz, *J*_gem_ = 12.9 Hz, H-3eq^*a*^), 2.50 (dd, 1H, *J*_3eq,4_ = 4.6 Hz, *J*_gem_ = 12.8 Hz, H-3eq^*b*^), 2.19–1.89 (m, 25H, 8Ac, H-3ax^*a*^), 1.64 (t, 1H, *J*_3ax,4_ = 12.8 Hz, H-3ax^*b*^), 1.46 (s, 3H, Ac), 1.04–0.99 (m, 2H, OCH_2_C*H_2_*Si), 0.01 (s, 9H, SiMe_3_); ^13^C NMR (125 MHz, CDCl_3_) δ 170.8, 170.6, 170.3, 170.2, 170.2, 170.0, 169.8, 168.5, 168.2, 167.6, 165.7, 165.5, 165.0, 139.0, 138.7, 138.6, 133.2, 132.9, 130.3, 130.0, 129.9, 129.9, 129.7, 129.4, 128.6, 128.4, 128.2, 128.2, 128.1, 128.0, 127.9, 127.4, 127.2, 127.2, 127.1, 126.9, 102.8, 100.7, 98.4, 96.9, 82.9, 81.9, 77.6, 74.9, 74.7, 74.4, 72.8, 72.0, 71.8, 71.7, 70.8, 69.1, 68.9, 68.7, 68.4, 68.2, 67.3, 67.1, 66.7, 63.7, 62.8, 62.1, 61.7, 53.2, 53.2, 49.3, 48.0, 37.5, 35.4, 29.7, 23.2, 21.4, 21.0, 20.8, 20.7, 20.7, 20.6, 20.4, 18.5, −1.5; HRMS (ESI) *m/z*: found [M + Na]^+^ 2009.6970, C_99_H_118_N_2_O_39_Si calcd for [M + Na]^+^ 2009.6973.

**2-(Trimethylsilyl)ethyl [methyl 4,7,8,9-tetra-*O*-acetyl-3,5-dideoxy-5-(methyl 5-acetamido-4,7,8,9-tetra-*O*-acetyl-3,5-dideoxy-d-*glycero*-α-d-*galacto*-2-nonulopyranosylonate)oxyacetamido-d-*glycero*-α-d-*galacto*-2-nonulopyranosylonate]-(2→3)-(2,4,6-tri-*O*-benzoyl-β-d-galactopyranosyl)-(1→4)-β-d-glucopyranoside (17)**. To a solution of **16** (48.8 mg, 24.0 µmol) in EtOAc (490 µL) was added Pd(OH)_2_/C (20%, 48.8 mg) at room temperature. After stirring for 2 h at room temperature under a hydrogen atmosphere as the reaction was monitored by TLC (30:1 CHCl_3_–MeOH, developed twice), the mixture was filtered through Celite. The filtrate was concentrated and the residue was purified by silica gel column chromatography (50:1 to 40:1 CHCl_3_–MeOH) to give **17** (39.8 mg, 97%): [α]_D_ +155.0° (c 0.1, CHCl_3_); ^1^H NMR (500 MHz, CDCl_3_) δ 8.34–7.42 (m, 15H, 3Ph), 5.97 (d, 1H, *J*_NH,5_ = 10.4 Hz, NH^*b*^), 5.87 (td, 1H, *J*_8,9a_ = 2.3 Hz, *J*_7,8_ = *J*_8,9b_ = 9.6 Hz, H-8^*b*^), 5.55 (dd, 1H, *J*_1,2_ = 8.0 Hz, *J*_2,3_ = 10.0 Hz, H-2^*c*^), 5.36–5.28 (m, 4H, H-7^*a*^, H-8^*a*^, H-4^*c*^, NH^*a*^), 5.07 (dd, 1H, *J*_6,7_ = 2.6 Hz, H-7^*b*^), 5.02 (d, 1H, H-1^*c*^), 4.96–4.89 (m, 2H, H-3^*c*^, H-4^*a*^), 4.82 (td, 1H, *J*_3eq,4_ = 4.4 Hz, *J*_3ax,4_ = *J*_4,5_ = 11.7 Hz, H-4^*b*^), 4.56–4.51 (m, 2H, OCH_2_, H-9a^*b*^), 4.38–4.25 (m, 5H, OCH_2_, H-9a^*a*^, H-1^*d*^, H-9b^*a*^, H-6a^*c*^), 4.15 (dd, 1H, *J*_6,7_ = 1.7 Hz, *J*_5,6_ = 10.7 Hz, H-6^*a*^), 4.11–4.04 (m, 3H, H-5^*c*^, H-5^*a*^, H-6b^*c*^), 3.98–3.76 (m, 10H, OC*H*_2_CH_2_Si, H-6a^*d*^, H-5^*b*^, 2OMe, H-9b^*b*^), 3.73–3.64 (m, 4H, H-6b^*d*^, H-4^*d*^, H-5^*d*^, H-6^*b*^), 3.57–3.50 (m, 2H, H-3^*d*^, OC*H*_2_CH_2_Si), 3.43 (t, 1H, *J*_1,2_ = *J*_2,3_ = 8.5 Hz, H-2^*d*^), 3.26 (d, 1H, *J*_OH,2_ = 9.4 Hz, OH-2^*d*^), 2.98 (s, 1H, OH-4^*d*^), 2.65 (dd, 1H, *J*_3eq,4_ = 4.6 Hz, *J*_gem_ = 12.8 Hz, H-3eq^*a*^), 2.56–2.53 (m, 2H, H-3eq^*b*^, OH-6^*d*^), 2.25–1.90 (m, 25H, 8Ac, H-3ax^*a*^), 1.63 (t, 1H, *J*_gem_ = 12.5 Hz, H-3ax^*b*^), 1.09–0.94 (m, 2H, OCH_2_C*H_2_*Si), 0.02 (s, 9H, SiMe_3_); ^13^C NMR (125 MHz, CDCl_3_) δ 172.3, 170.9, 170.8, 170.6, 170.2, 170.2, 169.9, 169.9, 168.5, 168.2, 167.5, 165.9, 165.7, 165.1, 133.5, 133.3, 133.2, 130.5, 130.0, 129.9, 129.4, 129.1, 129.0, 128.6, 128.5, 128.2, 101.8, 101.6, 98.3, 96.8, 79.8, 77.6, 77.3, 77.0, 76.7, 74.5, 74.3, 73.8, 72.8, 71.7, 71.5, 71.2, 70.6, 68.8, 68.7, 68.4, 68.2, 67.3, 67.3, 67.0, 66.5, 63.9, 63.6, 62.3, 62.1, 59.9, 53.3, 53.2, 49.2, 47.9, 37.4, 37.3, 31.9, 30.0, 29.6, 29.3, 27.0, 23.1, 22.6, 21.4, 21.0, 20.8, 20.8, 20.7, 20.6, 20.5, 18.1, 14.1, −1.5; HRMS (ESI) *m/z*: found [M + Na]^+^ 1739.5563, C_78_H_100_N_2_O_39_Si calcd for [M + Na]^+^ 1739.5565.

**2-(Trimethylsilyl)ethyl [5-(5-acetamido-3,5-dideoxy-d-*glycero*-α-d-*galacto*-2-nonulopyranosylonic acid)oxyacetamido-3,5-dideoxy-d-*glycero*-α-d-*galacto*-2-nonulopyranosylonic acid]-(2→3)-(β-d-galactopyranosyl)-(1→4)-β-d-glucopyranoside (3)**. To a solution of **17** (39.8 mg, 23.0 µmol) in pyridine (2.3 mL) was added lithium chloride (27.3 mg, 345 µmol) at room temperature. After stirring for 18 h under reflux as the reaction was monitored by TLC (10:1 CHCl_3_–MeOH, 4:1:0.1 CHCl_3_–MeOH–H_2_O), the reaction mixture was co-evaporated with toluene. The resulting residue was purified by gel filtration column chromatography (Sephadex LH-20) using MeOH as eluent. The product obtained was exposed to high vacuum and then dissolved in 0.1 M aq NaOH (250 µL). After stirring for 1 week at room temperature as the reaction was monitored by TLC (5:4:1 CHCl_3_–MeOH–H_2_O), the reaction mixture was neutralized with Dowex (H^+^) resin. The resin was filtered through cotton wool, and the filtrate was concentrated. The residue was purified by silica gel column chromatography (6:4:1 CHCl_3_–MeOH–H_2_O) followed by gel filtration column chromatography (Sephadex LH-20) using MeOH as eluent to give **3** (17.2 mg, 80%): [α]_D_ –0.5° (c 0.1, MeOH); ^1^H NMR (500 MHz, CD_3_OD) δ 4.50 (d, 1H, *J*_1,2_ = 7.8 Hz, H-1^*c*^), 4.39 (d, 1H, *J*_1,2_ = 7.9 Hz, H-1^*d*^), 4.32 (d, 1H, *J*_gem_ = 15.6 Hz, OCH_2_), 4.13 (d, 1H, OCH_2_), 4.09 (dd, 1H, *J*_3,4_ = 3.0 Hz, *J*_2,3_ = 9.8 Hz, H-3^*c*^), 4.01 (td, 1H, OC*H*_2_CH_2_Si), 3.96–3.48 (m, 25H, H-4^*a*^, H-5^*a*^, H-6^*a*^, H-7^*a*^, H-8^*a*^, H-9a^*a*^, H-9b^*a*^, H-4^*b*^, H-5^*b*^, H-6^*b*^, H-7^*b*^, H-8^*b*^, H-9a^*b*^, H-9b^*b*^, H-2^*c*^, H-4^*c*^, H-5^*c*^, H-6a^*c*^, H-6b^*c*^, H-2^*d*^, H-4^*d*^, H-5^*d*^, H-6a^*d*^, H-6b^*d*^, OC*H*_2_CH_2_Si), 3.27 (t, 1H, *J*_2,3_ = *J*_3,4_ = 7.9 Hz, H-3^*d*^), 2.81 (m, 2H, H-3eq^*a*^, H-3eq^*b*^), 2.04 (s, 3H, Ac), 1.79 (m, 2H, H-3ax^*a*^, H-3ax^*b*^), 1.08 (td, 1H, OCH_2_C*H*_2_Si), 0.98 (td, 1H, OCH_2_C*H*_2_Si), 0.03 (s, 9H, SiMe_3_); ^13^C NMR (125 MHz, CD_3_OD) δ 175.9, 175.0, 174.4, 173.8, 104.3, 103.2, 101.5, 101.0, 80.1, 77.1, 76.6, 76.1, 76.0, 74.3, 74.2, 74.0, 72.7, 69.7, 69.3, 68.7, 64.2, 64.0, 53.4, 49.8, 49.7, 49.6, 49.5, 49.5, 49.1, 41.2, 22.8, 18.9, −1.5; HRMS (ESI) *m/z*: found [M − 2H + Na]^−^ 1061.3476, C_39_H_66_N_2_O_28_Si calcd for [M − 2H + Na]^−^ 1061.3475.

**2-(Trimethylsilyl)ethyl [methyl 4,7,8,9-tetra-*O*-acetyl-3,5-dideoxy-5-(2,2,2-trichloroethoxycarbamoyl)-d-*glycero*-α-d-*galacto*-2-nonulopyranosylonate]-(2→3)-2,6-di-*O*-benzyl-β-d-galactopyranoside (20)**. To a mixture of **18** (100 mg, 218 µmol) and **19** (157 mg, 218 µmol) in propionitrile (2.2 mL) were added 3 Å molecular sieves (325 mg) and NIS (75.4 mg, 336 µmol) at room temperature. After stirring for 1 h, the mixture was cooled to −50 °C. TESOTf (7.5 µL, 3.3 µmol) was then added to the mixture at −50 °C. After stirring for 3.5 h at −50 °C as the reaction was monitored by TLC (1:1 EtOAc–*n*-hexane), the solution was diluted with CHCl_3_ and filtered through Celite. The filtrate was then washed with satd. aq. Na_2_S_2_O_3_, satd. aq. NaHCO_3_ and brine. The organic layer was subsequently dried over Na_2_SO_4_, and concentrated. The residue was purified by silica gel column chromatography (1:7 EtOAc–toluene) to give **20** (107 mg, 46%) and its β-isomer (4 mg, 2%): α-isomer: [α]_D_ –8.0° (c 0.1, CHCl_3_); ^1^H NMR (400 MHz, CDCl_3_) δ 7.42–7.23 (m, 10H, 2Ph), 5.40 (m, 1H, H-8^*b*^), 5.36 (dd, 1H, *J*_6,7_ = 2.0 Hz, *J*_7,8_ = 8.6 Hz, H-7^*b*^), 4.95 (td, 1H, *J*_3eq,4_ = 4.8 Hz, *J*_3ax,4_ = *J*_4,5_ = 11.5 Hz, H-4^*b*^), 4.87 (d, 1H, *J*_gem_ = 12.4 Hz, OCH_2_), 4.84 (d, 1H, *J*_gem_ = 11.7 Hz, OCH_2_), 4.79 (d, 1H, *J*_NH,5_ = 10.3 Hz, NH^*b*^), 4.71 (d, 1H, OCH_2_), 4.57 (s, 2H, OCH_2_), 4.47 (d, 1H, OCH_2_), 4.43 (d, 1H, *J*_1,2_ = 7.6 Hz, H-1^*c*^), 4.26 (dd, 1H, *J*_8,9a_ = 2.8 Hz, *J*_gem_ = 12.7 Hz, H-9a^*b*^), 4.13–4.08 (m, 2H, H-3^*c*^, H-6^*b*^), 4.04–3.99 (m, 2H, OC*H*_2_CH_2_Si, H-9b^*b*^), 3.81–3.58 (m, 9H, H-5^*b*^, H-4^*c*^, H-5^*c*^, H-6a^*c*^, H-6b^*c*^, OC*H*_2_CH_2_Si, OMe), 3.51 (dd, 1H, *J*_2,3_ = 9.7 Hz, H-2^*c*^), 2.72 (m, 2H, H-3eq^*b*^, OH-4^*c*^), 2.09–1.92 (m, 13H, 4Ac, H-3ax^*b*^), 1.02 (t, 2H, OCH_2_C*H_2_*Si), 0.00 (s, 9H, SiMe_3_); ^13^C NMR (100 MHz, CDCl_3_) δ 170.6, 170.3, 169.9, 168.5, 154.1, 139.1, 138.2, 128.4, 128.1, 127.8, 127.6, 127.6, 103.2, 97.7, 95.3, 77.6, 75.7, 74.5, 73.5, 72.6, 72.2, 69.3, 68.5, 68.3, 68.2, 67.4, 67.3, 62.2, 53.1, 51.5, 37.0, 21.1, 20.8, 20.6, 18.5, −1.4; MALDI *m/z*: found [M + Na]^+^ 1088.32, C_46_H_62_Cl_3_NO_19_Si calcd for [M + Na]^+^ 1088.16.

**2-(Trimethylsilyl)ethyl (methyl 4,7,8,9-tetra-*O*-acetyl-5-amino-3,5-dideoxy-d-*glycero*-α-d-*galacto*-2-nonulopyranosylonate)-(2→3)-2,6-di-*O*-benzyl-β-d-galactopyranoside (21)**. To a solution of **20** (20.0 mg, 18.7 µmol) in acetonitrile/AcOH (4:1, 1.3 mL) was added zinc powder (100 mg) at room temperature. After stirring for 40 min at room temperature as the reaction was monitored by TLC (15:1 CHCl_3_–MeOH), the mixture was filtered through Celite. The filtrate was concentrated, and the resulting residue was purified by silica gel column chromatography (60:1 CHCl_3_–MeOH) to give **21** (15.2 mg, 92%): [α]_D_ –11.5° (c 1.0, CHCl_3_); ^1^H NMR (500 MHz, CDCl_3_) δ 7.39–7.26 (m, 10H, 2Ph), 5.45 (s, 2H, H-7^*b*^, H-8^*b*^), 4.82 (d, 1H, *J*_gem_ = 11.5 Hz, PhC*H*_2_), 4.70 (d, 1H, PhC*H*_2_), 4.64–4.58 (m, 3H, H-4^*b*^, PhC*H_2_*), 4.42 (d, 1H, *J*_1,2_ = 7.8 Hz, H-1^*c*^), 4.29 (m, 1H, H-9a^*b*^), 4.12 (m, 1H, H-9b^*b*^), 4.08–4.00 (m, 2H, H-3^*c*^, OC*H*_2_CH_2_Si), 3.83–3.72 (m, 7H, H-4^*c*^, OMe, H-6a^*c*^, H-6^*b*^, H-6b^*c*^), 3.63–3.58 (m, 2H, H-5^*c*^, OC*H*_2_CH_2_Si), 3.51 (dd, 1H, *J*_2,3_ = 9.5 Hz, H-2^*c*^), 2.66 (dd, 1H, *J*_3eq,4_ = 4.6 Hz, *J*_gem_ = 12.9 Hz, H-3eq^*b*^), 2.59–2.53 (m, 2H, OH-4^*c*^, H-5^*b*^), 2.11, 2.04 and 1.93 (3s, 12H, 4Ac), 1.80 (t, 1H, *J*_3ax,4_ = 12.9 Hz, H-3ax^*b*^), 1.05–1.01 (m, 2H, OCH_2_C*H_2_*Si), 0.00 (s, 9H, SiMe_3_); ^13^C NMR (125 MHz, CDCl_3_) δ 170.6, 170.6, 170.2, 169.9, 168.5, 139.1, 138.2, 128.3, 128.1, 127.9, 127.6, 127.6, 127.4, 103.2, 98.0, 77.6, 77.6, 75.6, 75.1, 74.9, 73.5, 72.8, 71.9, 69.3, 68.4, 68.1, 67.9, 67.3, 62.1, 52.9, 51.0, 36.3, 29.7, 21.1, 21.0, 20.7, 20.5, 18.5, −1.4; HRMS (ESI) *m/z*: found [M + Na]^+^ 914.3604, C_43_H_61_NO_17_Si calcd for [M + Na]^+^ 914.3601.

**2-(Trimethylsilyl)ethyl [methyl 4,7,8,9-tetra-*O*-acetyl-3,5-dideoxy-5-(methyl 4,7,9-tri-*O*-acetyl-5-diacetamido-3,5-dideoxy-8-*O*-methyl-d-*glycero*-α-d-*galacto*-2-nonulopyranosylonate)oxyacetamido-d-*glycero*-α-d-*galacto*-2-nonulopyranosylonate]-(2→3)-2,6-di-*O*-benzyl-β-d-galactopyranoside (23)**. To a mixture of **22** (86.2 mg, 153 μmol) and **21** (209 mg, 230 μmol) in acetonitrile (3.1 mL) were added EDC·HCl (52.8 mg, 275 µmol) and HOBt (10.3 mg, 76 μmol) at room temperature. After stirring for 5 h at room temperature as the reaction was monitored by TLC (15:1 CHCl_3_–MeOH), the mixture was diluted with EtOAc. The solution was then washed with 2 M HCl, satd. aq. NaHCO_3_ and brine. The organic layer was subsequently dried over Na_2_SO_4_, and concentrated. The resulting residue was purified by silica gel column chromatography (55:1 CHCl_3_–MeOH) to give **23** (199 mg, 91%): [α]_D_ −15.0° (c 0.1, CHCl_3_); ^1^H NMR (500 MHz, CDCl_3_) δ 7.42–7.24 (m, 10H, 2Ph), 6.21 (d, 1H, *J*_NH,5_ = 10.2 Hz, NH^*b*^), 5.61 (td, 1H, *J*_3eq,4_ = 5.5 Hz, *J*_3ax,4_ = *J*_4,5_ = 9.5 Hz, H-4^*a*^), 5.44 (td, 1H, *J*_8,9a_ = 2.6 Hz, *J*_7,8_ = *J*_8,9b_ = 8.3 Hz, H-8^*b*^), 5.25 (dd, 1H, *J*_6,7_ = 2.1 Hz, H-7^*b*^), 4.99 (dd, 1H, *J*_6,7_ = 1.4 Hz, *J*_5,6_ = 10.3 Hz, H-6^*a*^), 4.94 (dd, 1H, *J*_7,8_ = 8.3 Hz, H-7^*a*^), 4.90–4.84 (m, 2H, H-4^*b*^, PhC*H*_2_), 4.72 (d, 1H, *J*_gem_ = 11.8 Hz, PhC*H*_2_), 4.59 (2d, 2H, *J*_gem_ = 13.2 Hz, PhC*H_2_*), 4.45 (d, 1H, *J*_1,2_ = 7.7 Hz, H-1^*c*^), 4.32–4.25 (m, 3H, H-9a^*a*^, H-9a^*b*^, H-5^*a*^), 4.22–3.93 (m, 11H, OCH_2_, H-3^*c*^, H-5^*b*^, H-6^*b*^, OCH_2_, H-9b^*b*^, H-9b^*a*^, OC*H*_2_CH_2_Si, CO_2_Me), 3.81–3.59 (m, 9H, CO_2_Me, H-4^*c*^, H-6a^*c*^, H-6b^*c*^, H-5^*c*^, H-8^*a*^, OC*H*_2_CH_2_Si), 3.52 (dd, 1H, *J*_2,3_ = 9.5 Hz, H-2^*c*^), 3.45 (s, 3H, OMe), 2,89 (dd, 1H, *J*_gem_ = 13.2 Hz, H-3eq^*a*^), 2.62–2.57 (m, 2H, OH-4^*c*^, H-3eq^*b*^), 2.38–1.93 (m, 29H, 9Ac, H-3ax^*b*^, H-3ax^*a*^), 1.04–1.01 (m, 2H, OCH_2_C*H_2_*Si), 0.01 (s, 9H, SiMe_3_); ^13^C NMR (125 MHz, CDCl_3_) δ 174.2, 173.6, 170.8, 170.3, 169.9, 169.8, 169.0, 168.7, 167.6, 139.2, 138.2, 128.3, 128.1, 127.7, 127.6, 127.5, 127.3, 103.1, 98.7, 97.9, 77.7, 77.6, 77.2, 76.6, 75.7, 74.8, 73.5, 72.8, 72.6, 70.4, 69.3, 68.6, 68.5, 68.2, 68.0, 67.3, 67.0, 63.5, 62.4, 61.5, 58.1, 56.6, 53.2, 53.0, 48.5, 38.0, 37.0, 29.6, 28.0, 26.1, 22.6, 21.1, 20.9, 20.8, 20.7, 20.7, 20.7, 18.4, 14.1, −1.5; HRMS (ESI) *m/z*: found [M + Na]^+^ 1459.5345, C_66_H_92_N_2_O_31_Si calcd for [M + Na]^+^ 1459.5346.

**2-(Trimethylsilyl)ethyl [methyl 4,7,8,9-tetra-*O*-acetyl-3,5-dideoxy-5-(methyl 5-acetamido-4,7,9-tri-*O*-acetyl-3,5-dideoxy-8-*O*-methyl-d-*glycero*-α-d-*galacto*-2-nonulopyranosylonate)oxyacetamido-d-*glycero*-α-d-*galacto*-2-nonulopyranosylonate]-(2→3)-2,6-di-*O*-benzyl-β-d-galactopyranoside (24)**. To a solution of **23** (49.5 mg, 34.0 µmol) in THF (1.4 mL) was added hydrazine acetate (9.5 mg, 100 µmol) at 0 °C. After stirring for 5 h at room temperature as the reaction was monitored by TLC (15:1 CHCl_3_–MeOH), the mixture was diluted with EtOAc. The solution was then washed with 2 M HCl, H_2_O, satd. aq. NaHCO_3_ and brine. The organic layer was subsequently dried over Na_2_SO_4_, and concentrated. The resulting residue was purified by silica gel column chromatography (50:1 CHCl_3_–MeOH) to give **24** (46.9 mg, 99%): [α]_D_ −65.0° (c 0.1, CHCl_3_); ^1^H NMR (500 MHz, CDCl_3_) δ 7.42–7.24 (m, 10H, 2Ph), 6.23 (d, 1H, *J*_NH,5_ = 10.0 Hz, NH^*b*^), 5.44 (td, 1H, *J*_8,9a_ = 2.7 Hz, *J*_7,8_ = *J*_8,9b_ = 8.0 Hz, H-8^*b*^), 5.28 (d, 1H, *J*_NH,5_ = 10.0 Hz, NH^*a*^), 5.23 (dd, 1H, *J*_6,7_ = 2.1 Hz, H-7^*b*^), 5.13 (dd, 1H, *J*_6,7_ = 1.8 Hz, *J*_7,8_ = 9.3 Hz, H-7^*a*^), 5.01 (td, 1H, *J*_3eq,4_ = 4.8 Hz, *J*_3ax,4_ = *J*_4,5_ = 11.5 Hz, H-4^*a*^), 4.92–4.84 (m, 2H, H-4^*b*^, PhC*H*_2_), 4.73 (d, 1H, *J*_gem_ = 11.8 Hz, PhC*H*_2_), 4.59 (2d, 2H, *J*_gem_ = 13.5 Hz, PhC*H_2_*), 4.44 (d, 1H, *J*_1,2_ = 7.7 Hz, H-1^*c*^), 4.31 (dd, 1H, *J*_gem_ = 12.4 Hz, H-9a^*b*^), 4.25–3.90 (m, 12H, OCH_2_, H-9a^*a*^, H-3^*c*^, H-5^*b*^, H-5^*a*^, H-9b^*a*^, OC*H*_2_CH_2_Si, H-9b^*b*^, OCH_2_, CO_2_Me), 3.81–3.59 (m, 9H, CO_2_Me, H-4^*c*^, H-6a^*c*^, H-6b^*c*^, H-8^*a*^, H-5^*c*^, OC*H*_2_CH_2_Si), 3.54–3.49 (m, 4H, H-2^*c*^, OMe), 2.74 (dd, 1H, *J*_gem_ = 12.9 Hz, H-3eq^*a*^), 2.64 (br s, 1H, OH-4^*c*^), 2.59 (dd, 1H, *J*_3eq,4_ = 4.6 Hz, *J*_gem_ = 13.0 Hz, H-3eq^*b*^), 2.13–1.88 (m, 26H, 8Ac, H-3ax^*b*^, H-3ax^*a*^), 1.04–1.01 (m, 2H, OCH_2_C*H_2_*Si), 0.01 (s, 9H, SiMe_3_); ^13^C NMR (125 MHz, CDCl_3_) δ 170.8, 170.8, 170.5, 170.3, 170.2, 170.0, 169.8, 168.8, 168.7, 167.8, 139.1, 138.2, 128.3, 128.1, 127.7, 127.6, 127.5, 127.3, 103.1, 98.6, 98.0, 77.7, 77.6, 77.2, 75.9, 75.7, 74.8, 73.5, 72.8, 72.6, 72.6, 69.3, 69.1, 68.6, 68.4, 68.2, 68.0, 67.4, 67.3, 63.5, 62.5, 61.7, 58.4, 53.1, 53.0, 49.2, 48.7, 37.5, 36.9, 31.9, 29.6, 23.2, 22.6, 21.1, 20.8, 20.7, 20.7, 20.6, 18.4, 14.1, −1.5; HRMS (ESI) *m/z*: found [M + Na]^+^ 1417.5242, C_64_H_90_N_2_O_30_Si calcd for [M + Na]^+^ 1417.5240.

**2-(Trimethylsilyl)ethyl [5-(5-acetamido-3,5-dideoxy-8-*O*-methyl-d-*glycero*-α-d-*galacto*-2-nonulopyranosylonic acid)oxyacetamido-3,5-dideoxy-d-*glycero*-α-d-*galacto*-2-nonulopyranosylonic acid]-(2→3)-β-d-galactopyranoside (4)**. To a solution of **24** (46.9 mg, 33.0 µmol) in EtOAc (1.3 mL) was added Pd(OH)_2_/C (20%, 46.9 mg) at room temperature. After stirring for 1 h at room temperature under a hydrogen atmosphere as the reaction was monitored by TLC (15:1 CHCl_3_–MeOH), the mixture was filtered through Celite. The filtrate was concentrated and the crude residue obtained was exposed to high vacuum overnight. The resulting residue was then dissolved in pyridine (3.3 mL), and lithium chloride (27.3 mg, 495 µmol) was added at room temperature. After stirring for 42 h under reflux as the reaction was monitored by TLC (4:1:0.1 CHCl_3_–MeOH–AcOH), the reaction mixture was co-evaporated with toluene. The obtained residue was purified by gel filtration column chromatography (Sephadex LH-20) using MeOH as eluent. The product was exposed to high vacuum and then dissolved in 0.1 M aq NaOH (5.0 mL). After stirring for 2 h at room temperature as the reaction was monitored by TLC (6:4:1 CHCl_3_–MeOH–H_2_O), the reaction mixture was neutralized with Dowex (H^+^) resin. The resin was filtered through cotton wool, and the filtrate was then evaporated. The residue was purified by silica gel column chromatography (6:4:1 CHCl_3_–MeOH–H_2_O), followed by gel filtration column chromatography (Sephadex LH-20) using MeOH as eluent to give **4** (23.3 mg, 79%): [α]_D_ +75.0° (c 0.1, MeOH); ^1^H NMR (500 MHz, CD_3_OD) δ 4.52–3.31 (m, 28H, H-4^*a*^, H-5^*a*^, H-6^*a*^, H-7^*a*^, H-8^*a*^, H-9a^*a*^, H-9b^*a*^, H-4^*b*^, H-5^*b*^, H-6^*b*^, H-7^*b*^, H-8^*b*^, H-9a^*b*^, H-9b^*b*^, H-1^*c*^, H-2^*c*^, H-3^*c*^, H-4^*c*^, H-5^*c*^, H-6a^*c*^, H-6b^*c*^, OCH_2_, OC*H_2_*CH_2_Si, OMe), 2.88 (br d, 1H, H-3eq^*a*^), 2.86 (br d, 1H, H-3eq^*b*^), 2.02 (s, 3H, Ac), 1.76–1.74 (m, 2H, H-3ax^*a*^, H-3ax^*b*^), 1.09–0.94 (m, 2H, OCH_2_C*H_2_*Si), 0.07 (s, 9H, SiMe_3_); ^13^C NMR (125 MHz, CD_3_OD) δ 175.5, 175.3, 175.3, 175.2, 104.2, 81.4, 79.4, 78.0, 76.4, 74.7, 74.4, 73.1, 70.8, 69.3, 68.9, 68.0, 63.4, 62.6, 61.7, 59.1, 54.0, 53.7, 49.8, 49.7, 49.6, 49.5, 42.0, 30.7, 22.8, 19.1, −1.6; HRMS (ESI) *m/z*: found [M − 2H + Na]^−^ 913.3095, C_34_H_58_N_2_O_23_Si calcd for [M − 2H + Na]^−^ 913.3097.

**Methyl [2-(trimethylsilyl)ethyl 4,7,8,9-tetra-*O*-acetyl-3,5-dideoxy-5-(2,2,2-trichloroethoxycarbamoyl)-d-*glycero*-α-d-*galacto*-2-nonulopyranosid]onate (25)**. To a mixture of **18** (1.0 g, 1.39 mmol) and 2-(trimethylsilyl)ethanol (1.0 mL, 6.97 mmol) in propionitrile (14 mL) were added 3 Å molecular sieves (1.4 g) and NIS (470 mg, 2.08 mmol) at room temperature. After stirring for 1 h, the mixture was cooled to −40 °C. TfOH (18.3 µL, 210 µmol) was then added to the mixture at −40 °C. After stirring for 5 days at −40 °C as the reaction was monitored by TLC (1:5 EtOAc–toluene), the reaction was quenched by the addition of triethylamine. The solution was diluted with CHCl_3_ and filtered through Celite. The filtrate was then washed with satd. aq. Na_2_S_2_O_3_ and brine. The organic layer was subsequently dried over Na_2_SO_4_, and concentrated. The residue was purified by silica gel column chromatography (1:5 EtOAc–toluene) to give **25** (654 mg, 65%, α:β = 6.2:1): [α]_D_ –5.0° (c 1.0, CHCl_3_); ^1^H NMR (500 MHz, CDCl_3_) δ 5.39 (s, 2H, H-7, H-8), 4.96–4.84 (m, 3H, H-4, OCH_2_, NH), 4.48 (d, 1H, *J*_gem_ = 12.1 Hz, OCH_2_), 4.29 (dd, 1H, *J*_8,9a_ = 1.8 Hz, *J*_gem_ = 11.7 Hz, H-9a), 4.19–4.12 (m, 2H, H-6, H-9b), 3.89 (m, 1H, OC*H*_2_CH_2_Si), 3.80 (s, 3H, OMe), 3.64 (q, 1H, *J*_4,5_ = *J*_5,6_ = *J*_5,NH_ = 10.3 Hz, H-5), 3.34 (m, 1H, OC*H*_2_CH_2_Si), 2.64 (dd, 1H, *J*_3eq,4_ = 4.7 Hz, *J*_gem_ = 12.6 Hz, H-3eq), 2.14–2.01 (m, 12H, 4Ac), 1.87 (t, 1H, *J*_3ax,4_ = 12.6 Hz, H-3ax), 0.91–0.85 (m, 2H, OCH_2_C*H_2_*Si), 0.03 (s, 9H, SiMe_3_); ^13^C NMR (125 MHz, CDCl_3_) δ 170.6, 170.4, 170.1, 169.7, 168.3, 154.1, 98.4, 95.4, 74.5, 71.8, 68.6, 68.3, 67.5, 62.6, 62.2, 52.6, 51.8, 38.4, 21.0, 20.8, 20.8, 20.7, 18.0, −1.4; HRMS (ESI) *m/z*: found [M + Na]^+^ 746.1180, C_26_H_40_Cl_3_NO_14_Si calcd for [M + Na]^+^ 746.1176.

**Methyl [2-(trimethylsilyl)ethyl 4,7,8,9-tetra-*O*-acetyl-5-amino-3,5-dideoxy-d-*glycero*-α-d-*galacto*-2-nonulopyranosid]onate (26)**. To a solution of **25** (40.0 mg, 55.0 µmol) in acetonitrile/AcOH (4:1, 1.8 mL) was added zinc powder (200 mg) at room temperature. After stirring for 5 h at room temperature as the reaction was monitored by TLC (15:1 CHCl_3_–MeOH), the mixture was filtered through Celite. The filtrate was concentrated and the residue was purified by silica gel column chromatography (60:1 CHCl_3_–MeOH) to give **26** (27.0 mg, 90%): [α]_D_ –8.5° (c 1.0, CHCl_3_); ^1^H NMR (500 MHz, CDCl_3_) δ 5.39 (s, 2H, H-7, H-8), 4.96–4.84 (m, 3H, H-4, OCH_2_, NH), 4.48 (d, 1H, *J*_gem_ = 12.1 Hz, OCH_2_), 4.29 (dd, 1H, *J*_8,9a_ = 1.8 Hz, *J*_gem_ = 11.7 Hz, H-9a), 4.19–4.12 (m, 2H, H-6, H-9b), 3.90–3.87 (m, 1H, OC*H*_2_CH_2_Si), 3.80 (s, 3H, OMe), 3.64 (q, 1H, *J*_4,5_ = *J*_5,6_ = *J*_5,NH_ = 10.3 Hz, H-5), 3.35–3.32 (m, 1H, OC*H*_2_CH_2_Si), 2.64 (dd, 1H, *J*_3eq,4_ = 4.7 Hz, *J*_gem_ = 12.6 Hz, H-3eq), 2.14–2.01 (m, 12H, 4Ac), 1.87 (t, 1H, *J*_3ax,4_ = 12.6 Hz, H-3ax), 0.91–0.85 (m, 2H, OCH_2_C*H_2_*Si), 0.03 (s, 9H, SiMe_3_); ^13^C NMR (125 MHz, CDCl_3_) δ 170.8, 170.7, 170.3, 169.9, 168.2, 98.3, 74.6, 71.9, 68.0, 67.9, 62.3, 62.0, 52.4, 51.2, 37.8, 21.0, 20.8, 20.7, 17.9, –1.4; HRMS (ESI) *m/z*: found [M + Na]^+^ 572.2137, C_23_H_39_NO_12_Si calcd for [M + Na]^+^ 572.2134.

**2-(Trimethylsilyl)ethyl [methyl 4,7,8,9-tetra-*O*-acetyl-3,5-dideoxy-5-(methyl 4,7,9-tri-*O*-acetyl-5-diacetamido-3,5-dideoxy-8-*O*-methyl-d-*glycero*-α-d-*galacto*-2-nonulopyranosylonate)oxyacetamido-d-*glycero*-α-d-*galacto*-2-nonulopyranosid]onate (27)**. To a solution of **22** (50.0 mg, 76.0 μmol) and **26** (76.0 mg, 143 μmol) in acetonitrile (3.1 mL) were added EDC·HCl (26.0 mg, 137 μmol) and HOBt (5.0 mg, 38.0 μmol) at room temperature. After stirring for 5 h at room temperature as the reaction was monitored by TLC (15:1 CHCl_3_–MeOH), the mixture was diluted with EtOAc. The solution was then washed with 2 M HCl, satd. aq. NaHCO_3_ and brine. The organic layer was subsequently dried over Na_2_SO_4_, and concentrated. The resulting residue was purified by silica gel column chromatography (80:1 CHCl_3_–MeOH) to give **27** (66.0 mg, 79%): [α]_D_ –2.0° (c 1.0, CHCl_3_); ^1^H NMR (500 MHz, CDCl_3_) δ 6.20 (d, 1H, *J*_NH,5_ = 9.8 Hz, NH^*b*^), 5.62 (td, 1H, *J*_3eq,4_ = 5.6 Hz, *J*_3ax,4_ = *J*_4,5_ = 9.9 Hz, H-4^*a*^), 5.42 (td, 1H, *J*_8,9a_ = 2.8 Hz, *J*_7,8_ = *J*_8,9b_ = 8.4 Hz, H-8^*b*^), 5.26 (dd, 1H, *J*_6,7_ = 1.5 Hz, H-7^*b*^), 5.00 (dd, 1H, *J*_6,7_ = 1.5 Hz, *J*_5,6_ = 10.3 Hz, H-6^*a*^), 4.94 (dd, 1H, *J*_7,8_ = 8.4 Hz, H-7^*a*^), 4.86 (td, 1H, *J*_3eq,4_ = 5.6 Hz, *J*_3ax,4_ = *J*_4,5_ = 7.8 Hz, H-4^*b*^), 4.32–3.99 (m, 9H, H-9a^*b*^, H-9a^*a*^, OCH_2_, H-5^*a*^, H-5^*b*^, H-9b^*a*^, H-9b^*b*^, H-6^*b*^), 3.94–3.88 (m, 4H, CO_2_Me, OC*H*_2_CH_2_Si), 3.82 (s, 3H, CO_2_Me), 3.55 (td, 1H, *J*_8,9a_ = 2.9 Hz, *J*_8,9b_ = 4.2 Hz, H-8^*a*^), 3.46 (s, 3H, OMe), 3.32 (m, 1H, OC*H*_2_CH_2_Si), 2.90 (dd, 1H, *J*_gem_ = 13.2 Hz, H-3eq^*a*^), 2.63 (dd, 1H, *J*_gem_ = 12.6 Hz, H-3eq^*b*^), 2.38 and 2.31 (2s, 6H, 2Ac), 2.16–1.91 (m, 23H, 7Ac, H-3ax^*a*^, H-3ax^*b*^), 0.93–0.85 (m, 2H, OCH_2_C*H_2_*Si), 0.04 (s, 9H, SiMe_3_); ^13^C NMR (125 MHz, CDCl_3_) δ 174.2, 173.6, 170.8, 170.5, 170.4, 170.1, 169.9, 169.8, 169.7, 168.9, 168.5, 167.5, 98.6, 98.5, 77.2, 76.5, 72.4, 70.4, 68.7, 68.3, 68.0, 67.4, 67.0, 63.5, 62.6, 62.4, 61.4, 58.1, 56.6, 53.2, 52.9, 48.7, 38.3, 38.0, 29.6, 28.0, 26.1, 21.0, 20.9, 20.8, 20.8, 20.8, 20.7, 20.7, 17.9, −1.3, −1.7; HRMS (ESI) *m/z*: found [M + Na]^+^ 1117.3883, C_46_H_70_N_2_O_26_Si calcd for [M + Na]^+^ 1117.3884.

**Methyl [2-(trimethylsilyl)ethyl 4,7,8,9-tetra-*O*-acetyl-3,5-dideoxy-5-(methyl 5-acetamido-4,7,9-tri-*O*-acetyl-3,5-dideoxy-8-*O*-methyl-d-*glycero*-α-d-*galacto*-2-nonulopyranosylonate)oxyacetamido-d-*glycero*-α-d-*galacto*-2-nonulopyranosid]onate (28)**. To a solution of **27** (65.6 mg, 59.0 µmol) in THF (2.4 mL) was added hydrazine acetate (16.6 mg, 180 µmol) at 0 °C. After stirring for 3.5 h at room temperature as the reaction was monitored by TLC (15:1 CHCl_3_–MeOH), the mixture was diluted with EtOAc. The solution was then washed with 2 M HCl, H_2_O, satd. aq. NaHCO_3_ and brine. The organic layer was subsequently dried over Na_2_SO_4_, and concentrated. The resulting residue was purified by silica gel column chromatography (50:1 CHCl_3_–MeOH) to give **28** (61.3 mg, 99%): [α]_D_ −14.0° (c 1.0, CHCl_3_); ^1^H NMR (500 MHz, CDCl_3_) δ 6.23 (d, 1H, *J*_NH,5_ = 9.7 Hz, NH^*b*^), 5.42 (td, 1H, *J*_8,9a_ = 2.6 Hz, *J*_7,8_ = *J*_8,9b_ = 6.1 Hz, H-8^*b*^), 5.27–5.23 (m, 2H, NH^*a*^, H-7^*b*^), 5.13 (dd, 1H, *J*_6,7_ = 1.8 Hz, *J*_7,8_ = 9.3 Hz, H-7^*a*^), 5.01 (td, 1H, *J*_3eq,4_ = 4.6 Hz, *J*_3ax,4_ = *J*_4,5_ = 11.6 Hz, H-4^*a*^), 4.88 (td, 1H, *J*_3eq,4_ = 4.6 Hz, *J*_3ax,4_ = *J*_4,5_ = 10.0 Hz, H-4^*b*^), 4.31 (dd, 1H, *J*_gem_ = 12.4 Hz, H-9a^*b*^), 4.28–4.01 (m, 8H, H-9a^*a*^, OCH_2_, H-5^*b*^, H-6^*a*^, H-9b^*b*^, H-5^*a*^, H-9b^*a*^, H-6^*b*^), 3.94–3.88 (m, 5H, OCH_2_, CO_2_Me, OC*H*_2_CH_2_Si), 3.82 (s, 3H, CO_2_Me), 3.69 (td, 1H, *J*_8,9a_ = 3.2 Hz, *J*_8,9b_ = 9.3 Hz, H-8^*a*^), 3.49 (s, 3H, OMe), 3.32 (td, 1H, OC*H*_2_CH_2_Si), 2.74 (dd, 1H, *J*_gem_ = 12.9 Hz, H-3eq^*a*^), 2.63 (dd, 1H, *J*_gem_ = 12.8 Hz, H-3eq^*b*^), 2.15–1.89 (m, 26H, 8Ac, H-3ax^*a*^, H-3ax^*b*^), 0.96–0.84 (m, 2H, OCH_2_C*H_2_*Si), 0.03 (s, 9H, SiMe_3_); ^13^C NMR (125 MHz, CDCl_3_) δ 170.8, 170.8, 170.5, 170.4, 170.2, 169.8, 168.7, 168.6, 167.8, 98.6, 98.6, 77.2, 75.9, 72.6, 72.5, 69.1, 68.6, 68.5, 68.0, 67.8, 63.5, 62.6, 62.5, 61.7, 58.4, 53.1, 52.6, 49.2, 48.9, 38.3, 37.5, 29.6, 23.2, 21.0, 20.8, 20.8, 20.7, 17.9, −1.5; HRMS (ESI) *m/z*: found [M + Na]^+^ 1075.3772, C_44_H_68_N_2_O_25_Si calcd for [M + Na]^+^ 1075.3773.

**3,5-Dideoxy-[2-(trimethylsilyl)ethyl 5-acetamido-3,5-dideoxy-8-*O*-methyl-d-*glycero*-α-d-*galacto*-2-nonulopyranosylonic acid]oxyacetamido-d-*glycero*-α-d-*galacto*-2-nonulopyranosidonic acid (5)**. To a solution of **28** (61.3 mg, 58.0 µmol) in pyridine (5.8 mL) was added lithium chloride (36.9 mg, 870 µmol) at room temperature. After stirring for 22 h under reflux as the reaction was monitored by TLC (6:4:0.1 CHCl_3_–MeOH–AcOH), the reaction mixture was co-evaporated with toluene. The crude residue was purified by gel filtration column chromatography (Sephadex LH-20) using MeOH as eluent. The product was exposed to high vacuum and then dissolved in 0.1 M aq NaOH (5.0 mL). After stirring for 2 h at room temperature as the reaction was monitored by TLC (5:4:1 CHCl_3_–MeOH–H_2_O), the reaction mixture was neutralized with Dowex (H^+^) resin. The resin was filtered through cotton wool and the filtrate was then evaporated. The residue was purified by silica gel column chromatography (6:4:1 CHCl_3_–MeOH–H_2_O) followed by gel filtration column chromatography (Sephadex LH-20) using MeOH as eluent to give **5** (33.0 mg, 78%): [α]_D_ –11.5° (c 0.1, MeOH); ^1^H NMR (500 MHz, CD_3_OD) δ 4.31–3.35 (m, 21H, H-4^*a*^, H-5^*a*^, H-6^*a*^, H-7^*a*^, H-8^*a*^, H-9a^*a*^, H-9b^*a*^, H-4^*b*^, H-5^*b*^, H-6^*b*^, H-7^*b*^, H-8^*b*^, H-9a^*b*^, H-9b^*b*^, OCH_2_, OC*H_2_*CH_2_Si, OMe), 2.82 (dd, 1H, *J*_3eq,4_ = 4.2 Hz, *J*_gem_ = 12.5 Hz, H-3eq^*a*^), 2.62 (br d, 1H, H-3eq^*b*^), 1.99 (s, 3H, Ac), 1.72 (t, 1H, *J*_3ax,4_ = *J*_gem_ = 11.7 Hz, H-3ax^*b*^), 1.59 (t, 1H, *J*_3ax,4_ = 12.5 Hz, H-3ax^*a*^), 0.92–0.85 (m, 2H, OCH_2_C*H_2_*Si), 0.01 (s, 9H, SiMe_3_); ^13^C NMR (125 MHz, CD_3_OD) δ 175.0, 174.9, 174.2, 173.3, 101.6, 101.3, 81.4, 79.5, 79.4, 74.6, 74.0, 73.1, 70.0, 69.2, 69.0, 64.2, 62.5, 61.5, 58.9, 54.1, 54.0, 49.9, 49.7, 49.6, 49.3, 42.7, 41.3, 30.7, 22.7, −1.5; HRMS (ESI) *m/z*: found [M − 2H + Na]^−^ 751.2572, C_28_H_50_N_2_O_18_Si calcd for [M − 2H + Na]^−^ 751.2575.

**Methyl [2-(trimethylsilyl)ethyl 5-acetamido-4,7,9-tri-*O*-acetyl-3,5-dideoxy-d-*glycero*-α-d-*galacto*-2-nonulopyranosid]onate (29)**. To a solution of **25** (100 mg, 138 µmol) in DMF/AcOH (4:1, 4.6 mL) was added zinc powder (500 mg) at room temperature. After stirring for 12 h at room temperature as the reaction was monitored by TLC (15:1 CHCl_3_–MeOH), the mixture was filtered through Celite. The filtrate was concentrated and the residue was purified by silica gel column chromatography (60:1 CHCl_3_–MeOH) to give **29** (62.5 mg, 82%): [α]_D_ –12.5° (c 1.0, CHCl_3_); ^1^H NMR (500 MHz, CDCl_3_) δ 5.46 (d, 1H, *J*_NH,5_ = 10.2 Hz, NH), 5.13 (dd, 1H, *J*_6,7_ = 2.2 Hz, *J*_7,8_ = 8.7 Hz, H-7), 4.84 (td, 1H, *J*_3eq,4_ = 5.6 Hz, *J*_3ax,4_ = *J*_4,5_ = 7.3 Hz, H-4), 4.18–4.13 (m, 3H, H-9a, H-5, H-8), 4.09–4.05 (m, 2H, H-9b, OH), 3.91–3.86 (m, 5H, H-6, OMe, OC*H*_2_CH_2_Si), 3.44 (m, 1H, OC*H*_2_CH_2_Si), 2.70 (dd, 1H, *J*_gem_ = 13.0 Hz, H-3eq), 2.12–1.93 (m, 10H, 3Ac, H-3ax), 1.88 (s, 3H, Ac), 0.92–0.88 (m, 2H, OCH_2_C*H_2_*Si), 0.00 (s, 9H, SiMe_3_); ^13^C NMR (125 MHz, CDCl_3_) δ 170.9, 170.9, 170.3, 170.3, 169.8, 98.6, 77.2, 72.6, 69.5, 68.9, 68.3, 64.7, 62.2, 53.4, 49.0, 37.4, 23.0, 20.9, 20.8, 17.8, −1.4; HRMS (ESI) *m/z*: found [M + Na]^+^ 572.2137, C_23_H_39_NO_12_Si calcd for [M + Na]^+^ 572.2134.

**Methyl [2-(trimethylsilyl)ethyl 5-acetamido-4,7,9-tri-*O*-acetyl-8-*O*-chloroacetyl-3,5-dideoxy-d-*glycero*-α-d-*galacto*-2-nonulopyranosid]onate (30)**. To a solution of **29** (72.4 mg, 130 µmol) in THF (1.3 mL) were added chloroacetic anhydride (33.8 mg, 197 µmol) and DMAP (1.6 mg, 13.0 µmol) at 0 °C. After stirring for 12 h at room temperature as the reaction was monitored by TLC (20:1 CHCl_3_–MeOH), the reaction was quenched by the addition of MeOH at 0 °C. The residue was then diluted with EtOAc, which was subsequently washed with 2 M HCl, H_2_O, satd. aq. NaHCO_3_ and brine. The organic layer was dried over Na_2_SO_4_, and concentrated. The resulting residue was purified by silica gel column chromatography (60:1 CHCl_3_–MeOH) to give **30** (70.8 mg, quant.): [α]_D_ –10.0° (c 1.0, CHCl_3_); ^1^H NMR (500 MHz, CDCl_3_) δ 5.48 (m, 1H, H-8), 5.34 (dd, 1H, *J*_6,7_ = 1.6 Hz, *J*_7,8_ = 9.0 Hz, H-7), 5.26 (d, 1H, *J*_NH,5_ = 9.6 Hz, NH), 4.81 (td, 1H, *J*_3eq,4_ = 5.2 Hz, *J*_3ax,4_ = *J*_4,5_ = 7.8 Hz, H-4), 4.34–4.30 (m, 2H, H-9a, OCH_2_), 4.17 (d, 1H, *J*_gem_ = 15.0 Hz, OCH_2_), 4,12–4.07 (m, 3H, H-5, H-6, H-9b), 3.86 (m, 1H, OC*H*_2_CH_2_Si), 3.80 (s, 3H, OMe), 3,26 (m, 1H, OC*H*_2_CH_2_Si), 2.58 (dd, 1H, *J*_gem_ = 12.9 Hz, H-3eq), 2.15, 2.04 and 2.03 (3s, 9H, 3Ac), 1.95 (t, 1H, H-3ax), 1.88 (s, 3H, Ac), 0.94–0.82 (m, 2H, OCH_2_C*H_2_*Si), 0.03 (s, 9H, SiMe_3_); ^13^C NMR (125 MHz, CDCl_3_) δ 172.5, 172.0, 171.7, 170.2, 167.8, 100.0, 78.7, 78.7, 78.5, 78.2, 73.7, 71.3, 70.5, 68.4, 64.2, 63.6, 54.1, 50.8, 42.7, 39.7, 31.1, 24.6, 22.3, 22.3, 22.1, 19.4, −1.4; HRMS (ESI) *m/z*: found [M + Na]^+^ 648.1851, C_25_H_40_ClNO_13_Si calcd for [M + Na]^+^ 648.1850.

**Methyl [2-(trimethylsilyl)ethyl 4,7,9-tri-*O*-acetyl-8-*O*-chloroacetyl-5-diacetamido-3,5-dideoxy-d-*glycero*-α-d-*galacto*-2-nonulopyranosid]onate (31)**. To a solution of **30** (71.2 mg, 113 µmol) in isopropenyl acetate (4.5 mL) was added *p*-toluenesulfonic acid monohydrate (2.0 mg, 11.3 µmol) at room temperature. After stirring for 7 h at 85 °C as the reaction was monitored by TLC (20:1 CHCl_3_–MeOH), the reaction was quenched by the addition of triethylamine. The residue was then diluted with EtOAc, and washed with H_2_O and brine. The organic layer was subsequently dried over Na_2_SO_4_, and concentrated. The resulting residue was purified by silica gel column chromatography (100:1 CHCl_3_–MeOH) to give **31** (75.5 mg, quant.): [α]_D_ +9.0° (c 1.0, CHCl_3_); ^1^H NMR (500 MHz, CDCl_3_) δ 5.48–5.43 (m, 2H, H-8, H-4), 5.16 (dd, 1H, *J*_6,7_ = 1.9 Hz, *J*_7,8_ = 8.7 Hz, H-7), 4.99 (dd, 1H, *J*_5,6_ = 10.1 Hz, H-6), 4.35 (d, 1H, *J*_gem_ = 15.1 Hz, OCH_2_), 4.31 (dd, 1H, *J*_8,9a_ = 2.6 Hz, *J*_gem_ = 12.7 Hz, H-9a), 4.21 (t, 1H, *J*_4,5_ = 10.1 Hz, H-5), 4.16–4.12 (m, 2H, OCH_2_, H-9b), 3.89 (m, 1H, OC*H*_2_CH_2_Si), 3.83 (s, 3H, OMe), 3.44 (m, 1H, OC*H*_2_CH_2_Si), 2.76 (dd, 1H, *J*_3eq,4_ = 5.2 Hz, *J*_gem_ = 12.9 Hz, H-3eq), 2.38–1.98 (m, 15H, 5Ac), 1.84 (t, 1H, *J*_3ax,4_ = 12.9 Hz, H-3ax), 0.90–0.85 (m, 2H, OCH_2_C*H_2_*Si), 0.03 (s, 9H, SiMe_3_); ^13^C NMR (125 MHz, CDCl_3_) δ 174.4, 173.5, 170.5, 169.6, 167.9, 166.4, 98.5, 77.2, 69.8, 69.3, 66.8, 66.7, 62.5, 61.6, 56.9, 52.7, 41.2, 39.3, 27.9, 26.0, 20.9, 20.7, 20.6, 17.9, −1.4; HRMS (ESI) *m/z*: found [M + Na]^+^ 690.1956, C_27_H_42_ClNO_14_Si calcd for [M + Na]^+^ 690.1955.

**Methyl [2-(trimethylsilyl)ethyl 4,7,9-tri-*O*-acetyl-5-diacetamido-3,5-dideoxy-8-*O*-methyl-d-*glycero*-α-d-*galacto*-2-nonulopyranosid]onate (32)**. To a solution of **31** (79.5 mg, 118 µmol) in DMF (4.5 mL) were added 1-selenocarbamoylpiperidine (45.4 mg, 238 µmol) and 2,6-lutidine (20 µL, 177 µmol) at room temperature. After stirring for 1 h at 65 °C as the reaction was monitored by TLC (1:3 EtOAc–*n*-hexane), the mixture was diluted with EtOAc. The solution was then washed with 2 M HCl, H_2_O, satd. aq. NaHCO_3_ and brine. The organic layer was subsequently dried over Na_2_SO_4_, and concentrated. The crude residue was exposed to high vacuum. The residue was then dissolved in CH_2_Cl_2_ (4.7 mL). Trimethyloxonium tetrafluoroborate (87.3 mg, 590 µmol) and 2,4,6-tri-*tert*-butylpyrimidine (161 mg, 650 µmol) were added to the mixture at room temperature. After stirring for 4 h under reflux as the reaction was monitored by TLC (1:3 EtOAc–*n*-hexane), the reaction was quenched by the addition of iced water. The residue was then diluted with EtOAc, and washed with H_2_O, satd. aq. NaHCO_3_ and brine. The organic layer was subsequently dried over Na_2_SO_4_, and concentrated. The resulting residue was purified by silica gel column chromatography (1:3 EtOAc–*n*-hexane) to give **32** (55.6 mg, 78%): [α]_D_ +5.0° (c 1.0, CHCl_3_); ^1^H NMR (500 MHz, CDCl_3_) δ 5.56 (td, 1H, *J*_3eq,4_ = 5.4 Hz, *J*_3ax,4_ = *J*_4,5_ = 10.4 Hz, H-4), 4.99 (dd, 1H, *J*_6,7_ = 1.4 Hz, *J*_7,8_ = 7.5 Hz, H-7), 4.94 (dd, 1H, *J*_5,6_ = 10.4 Hz, H-6), 4.34 (dd, 1H, *J*_8,9a_ = 1.4 Hz, *J*_gem_ = 10.1 Hz, H-9a), 4.15 (t, 1H, H-5), 4.09 (dd, 1H, *J*_8,9b_ = 5.0 Hz, H-9b), 3.93 (m, 1H, OC*H*_2_CH_2_Si), 3.87 (s, 3H, CO_2_Me), 3.76 (m, 1H, OC*H*_2_CH_2_Si), 3.47 (s, 3H, OMe), 2.80 (dd, 1H, *J*_gem_ = 13.0 Hz, H-3eq), 2.36–1.99 (m, 15H, 5Ac), 1.85 (near t, 1H, H-3ax), 0.93–0.89 (m, 2H, OCH_2_C*H_2_*Si), 0.03 (s, 9H, SiMe_3_); ^13^C NMR (125 MHz, CDCl_3_) δ 174.4, 173.8, 170.8, 170.1, 169.7, 168.0, 98.7, 69.9, 68.3, 67.1, 62.0, 61.9, 58.1, 57.4, 52.6, 38.7, 28.0, 25.9, 20.9, 20.8, 20.8, 18.0, −1.5; HRMS (ESI) *m/z*: found [M + Na]^+^ 628.2398, C_26_H_43_NO_13_Si calcd for [M + Na]^+^ 628.2396.

**Methyl [2-(trimethylsilyl)ethyl 5-acetamido-4,7,9-tri-*O*-acetyl-3,5-dideoxy-8-*O*-methyl-d-*glycero*-α-d-*galacto*-2-nonulopyranosid]onate (33)**. To a solution of **32** (46.7 mg, 77.0 µmol) in THF (3.1 mL) was added hydrazine acetate (21.3 mg, 230 µmol) at 0 °C. After stirring for 4 h at room temperature as the reaction was monitored by TLC (20:1 CHCl_3_–MeOH), the mixture was diluted with EtOAc. The solution was then washed with 2 M HCl, H_2_O, satd. aq. NaHCO_3_ and brine. The organic layer was subsequently dried over Na_2_SO_4_, and concentrated. The resulting residue was purified by silica gel column chromatography (60:1 CHCl_3_–MeOH) to give **33** (34.8 mg, 80%): [α]_D_ –12.0° (c 1.0, CHCl_3_); ^1^H NMR (500 MHz, CDCl_3_) δ 5.15–5.13 (m, 2H, H-7, NH), 4.92 (td, 1H, *J*_3eq,4_ = 5.0 Hz, *J*_3ax,4_ = *J*_4,5_ = 12.5 Hz, H-4), 4.26 (dd, 1H, *J*_8,9a_ = 3.3 Hz, *J*_gem_ = 12.3 Hz, H-9a), 4.11–4.08 (m, 2H, H-9b, H-6), 4.03 (t, 1H, *J*_5,NH_ = 12.5 Hz, H-5), 3.91 (m, 1H, OC*H*_2_CH_2_Si), 3.83 (s, 3H, CO_2_Me), 3.77 (m, 1H, H-8), 3.54–3.49 (m, 4H, OMe, OC*H*_2_CH_2_Si), 2.62 (dd, 1H, *J*_gem_ = 12.5 Hz, H-3eq), 2.15–1.97 (m, 9H, 3Ac), 1.91 (t, 1H, H-3ax), 1.75 (s, 3H, Ac), 0.95–0.88 (m, 2H, OCH_2_C*H_2_*Si), 0.03 (s, 9H, SiMe_3_); ^13^C NMR (125 MHz, CDCl_3_) δ 170.9, 170.8, 170.1, 170.0, 168.5, 98.7, 77.6, 76.2, 72.1, 69.4, 68.3, 62.0, 61.8, 58.4, 52.5, 49.5, 37.7, 29.7, 23.2, 20.9, 20.9, 20.8, 18.0, −1.6; HRMS (ESI) *m/z*: found [M + Na]^+^ 586.2292, C_24_H_41_NO_12_Si calcd for [M + Na]^+^ 586.2290.

**2-(Trimethylsilyl)ethyl 5-acetamido-3,5-dideoxy-8-*O*-methyl-d-*glycero*-α-d-*galacto*-2-nonulopyranosidonic acid (6)**. To a solution of **33** (34.8 mg, 61.0 µmol) in pyridine (6.2 mL) was added lithium chloride (39.2 mg, 930 µmol) at room temperature. After stirring for 22 h under reflux as the reaction was monitored by TLC (6:4:0.1 CHCl_3_–MeOH–AcOH), the reaction mixture was co-evaporated with toluene. The crude residue was purified by gel filtration column chromatography (Sephadex LH-20) using MeOH as eluent. The product was exposed to high vacuum, and then dissolved in 0.1 M aq NaOH (5.0 mL). After stirring for 2 h at room temperature as the reaction was monitored by TLC (5:4:1 CHCl_3_–MeOH–H_2_O), the reaction mixture was neutralized with Dowex (H^+^) resin. The resin was filtered through cotton wool and the filtrate was then evaporated. The residue was purified by silica gel column chromatography (6:4:1 CHCl_3_–MeOH–H_2_O) followed by gel filtration column chromatography (Sephadex LH-20) using MeOH as eluent to give **6** (12.3 mg, 48%): [α]_D_ −3.0° (c 1.0, MeOH); ^1^H NMR (500 MHz, D_2_O) δ 3.98 (dd, 1H, *J*_8,9a_ = 2.5 Hz, *J*_gem_ = 12.0 Hz, H-9a), 3.91–3.83 (m, 3H, OC*H*_2_CH_2_Si, H-7, H-5), 3.74–3.59 (m, 4H, H-4, H-9b, OC*H*_2_CH_2_Si, H-6), 3.48–3.44 (m, 4H, H-8, OMe), 2.65 (dd, 1H, *J*_3eq,4_ = 4.5 Hz, *J*_gem_ = 12.5 Hz, H-3eq), 2.02 (s, 3H, Ac), 1.69 (t, 1H, *J*_3ax,4_ = 12.5 Hz, H-3ax), 0.96–0.92 (m, 2H, OCH_2_C*H_2_*Si), 0.01 (s, 9H, SiMe_3_); ^13^C NMR (125 MHz, CD_3_OD) δ 175.1, 171.3, 100.3, 81.4, 74.5, 69.2, 68.8, 62.7, 61.2, 58.5, 54.1, 49.6, 49.5, 49.3, 49.1, 48.5, 42.2, 22.7, 19.1, −1.3; HRMS (ESI) *m/z*: found [M − H]^−^ 422.1851, C_17_H_33_NO_9_Si calcd for [M − H]^−^ 422.1852.

### 3.2. Materials Used in Biological Experiments

PC12 cell was obtained from the RIKEN Cell Bank (Tsukuba, Japan). The human neuroblastoma cell line SH-SY5Y (ATCC CRL-2266) was obtained from American Type Culture Collection (Manassas, VA, USA). RPMI 1640 medium was purchased from Life Technologies Japan Ltd. (Tokyo, Japan). Dulbecco’s Modified Eagle’s Medium/Nutrient Mixture F-12 Ham (DMEM/F12) and Toluidine blue-O were purchased from Sigma-Aldrich (St. Louis, MO, USA,). Fetal bovine serum (FBS) and horse serum (HS) were purchased from PAA Laboratories GmbH (Pasching, Austria). U0126 and Phos-tag acrylamide AAL-107 were obtained from Wako Pure Chemical Industries (Osaka, Japan). Neuronal growth factor (NGF) was purchased from Alomone Labs Ltd. (Jerusalem, Israel). Protease inhibitor cocktail (containing AEBSF, aprotinin, E-64, leupeptin, EDTA, bestatin and pepstatin A), phosphatase inhibitor cocktail (containing imidazole, EDTA, Na_3_VO_4_, β-glycerophosphoric acid, sodium (+)-tartrate, NaF and Na_2_MoO_4_), and transparent 96-well microplates precoated with poly l-lysin were purchased from Nacalai Tesque (Kyoto, Japan). Anti-p44/42 MAPK (ERK 1/2) rabbit monoclonal antibody 137F5, anti-Akt (pan) (11E7) rabbit monoclonal antibody and anti-phospho-Akt (Ser 473) rabbit monoclonal antibody were obtained from Cell Signaling Technology (Danvers, MA, USA). Anti-actin (c-2) mouse monoclonal antibody was purchased from Santa Cruz Biotechnology (Dallas, TX, USA).

### 3.3. Neurite Outgrowth Evaluation

#### 3.3.1. PC12 Cells

Cells were plated onto transparent 96-well microplates precoated with poly l-lysin with 1 × 10^4^ cells per well and cultured with RPMI 1640 medium supplemented with 5% heat-inactivated FBS and 10% heat-inactivated HS in 5% CO_2_ at 37 °C. After 24 h, cells were washed with fresh RPMI 1640 and the medium was replaced by 200 µL/well of RPMI medium with low sera (0.05% heat-inactivated FBS and 0.1% heat-inactivated HS) supplemented with synthesized saccharides and 1 or 5 ng/mL of NGF for neurite outgrowth evaluation. After 3 days, medium was changed and the cells were cultured for more 2 days. Cells were fixed with 4% paraformaldehyde in PBS for 30 min and stained by Toluidine blue-O in citrate buffer (pH 4.0) for 10 min. After brief wash with MilliQ water, morphological changes were observed and photographed with inverted microscope (IX70, Olympus, Tokyo, Japan) with a CCD camera (DP21, Olympus). For the analysis of morphological changes, four random areas were selected per well and photographed. The length of neurite and cell body in the image were quantified by ImageJ software (National Institutes of Health, Bethesda, MD, USA). Measurements were performed on triplicate. A sufficient number of parameters were acquired for the analysis of at least 150 cells. Mean total of neurite lengths was calculated at each cell.

#### 3.3.2. SH-SY5Y Cells

SH-SY5Y cells were seeded in 96-well culture plates at a density of 1 x 10^4^ cells/well with DMEM/F12 supplemented with 10% FBS and incubated overnight in a 5% CO_2_ incubator at 37 °C. After washing with 200 µL/well of fresh DMEM/F12, cells were incubated with 200 µL/well of various concentrations of the trisaccharide **4** diluted in DMEM/F12 containing 1% FBS for 72 h, with or without MEK inhibitor U0126. The evaluation of cells by photographs was done as well as PC-12 cells. A sufficient number of parameters were acquired for the analysis of at least 90 cells. In the case of MEK inhibitor U0126, 180 cells were measured. Mean total neurite length per cell of 90 or 180 cells was calculated at each dose of the trisaccharide **4**.

### 3.4. Analysis of Cell Signaling Cascade

#### 3.4.1. Sample Preparation

SH-SY5Y cells were seeded in 12-well culture plates at a density of 8 × 10^5^ cells/well with DMEM/F12 supplemented with 10% FBS and incubated overnight in a 5% CO_2_ incubator at 37 °C. After washing with 2 mL/well of fresh DMEM/F12 twice, cells were pre-incubated with 1.8 mL/well of fresh DMEM/F12 for 1 h. The time-course of ERK 1/2 and Akt phosphorylation was analyzed as follows: 0.2 mL/well of the trisaccharide **4** diluted in DMEM/F12 was added to each well (final concentration: 1 nM or 10 nM) and incubated for 0 to 60 min. As positive control, NGF (final concentration: 40 ng/mL) was used. For the analysis of dose-dependency, 0.2 mL/well of the trisaccharide **4** (final concentration: 0 to 100 nM) was added to wells and cells were incubated for 5 min.

The trisaccharide **4**-stimulated ERK 1/2 phosphorylation was confirmed in the presence of MEK inhibitor U0126. In brief, cells were pre-incubated with 1.8 mL/well of DMEM/F12 containing U0126 for 1 h, followed by adding 0.2 mL/well of the trisaccharide **4** (final concentration: 1 nM) for 5 min.

After incubation, cells were washed with 2 mL/well of ice-cold PBS and treated with 75 µL/well of ice-cold cell lysis buffer (50 mM Tris-HCl, pH 7.6, 1% Triton X-100, 150 mM NaCl supplemented with protease inhibitor cocktail and phosphatase inhibitor cocktail) for 30 min on ice. Cell lysates were collected into microtubes, centrifuged for 5 min at 10,000× g at 4 °C, and supernatants were treated with 5-fold sample buffer (10% SDS, 40% glycerol, 25% 2-mercaptoethanol in 325 mM Tris-HCl, pH 6.9) for 5 min at 100 °C.

#### 3.4.2. ECL-Western Blotting

ERK 1/2 phosphorylation was investigated by Phos-tag SDS-PAGE according to the method of Kinoshita *et al.* [54,55]. In brief, 5-fold sample buffer treated cell lysates were loaded onto 10% acrylamide gel containing 15 µM Phos-tag acrylamide, 0.05 mM Zn(NO_3_)_2_, 0.1% SDS, 357 mM Bis-Tris (pH 6.8) and electrophoresed in MOPS buffer (0.1% SDS, 0.1 M Tris, 0.1 M MOPS, 5 mM NaHSO_3_, pH 7.8) at 20 mA/gel. In the analysis of Akt phosphorylation, samples were loaded on 7.5% separation gel and resolved by Laemmli SDS-PAGE. After electrophoresis, separated proteins were electrotransferred to PVDF membranes. The membranes were blocked by blocking solution (1% BSA in Tris-buffered saline-0.1% tween 20 (TBS-T)) for 1 h at room temperature and incubated with anti-p44/42 MAPK (ERK1/2) antibody, anti-Akt (pan) antibody, anti-phospho-Akt (Ser607) antibody or anti-actin antibody diluted in blocking solution at 4 °C overnight. After washing with TBS-T, the membranes were incubated with HRP-labeled anti-rabbit IgG antibody or HRP-labeled anti-mouse IgG antibody (BIO-RAD, Hercules, CA, USA) diluted in blocking buffer for 1 h at room temperature and the chemiluminescent signal was detected using LAS3000mini (Fujifilm, Tokyo, Japan).

The expression levels of ERK 1/2 (ERK), phosphorylated ERK 1/2 (p-ERK), Akt, and phosphorylated Akt (p-Akt) were quantified by densitometric analysis using ImageJ and results were expressed as the ratio of phosphorylated forms (p-ERK or p-Akt) to non-phosphorylated forms (ERK or Akt). The expression level of Akt and p-Akt was normalized by beta-actin.

### 3.5. Statistical Analysis

All data were evaluated by Dunnett’s test or Student’s *t*-test using IBM SPSS Statistics version 19 (IBM Company, Armonk, NY, USA).

## 4. Conclusions

Recently, we reported the first total synthesis of ganglioside LLG-3, which was originally identified in the starfish *Linckia laevigata* [36,56], and showed that synthetic LLG-3 could stimulate neuritogenesis in rat PC12 cells [40]. In this report, we investigated the neuritogenic potential of the glycan moiety of LLG-3. We evaluated LLG-3 glycan analogues **2**–**6** by a SAR study and identified trisaccharide **4**, which was the trisaccharide at the non-reducing end of the tetrasaccharide part of the LLG-3 ganglioside, as the minimum motif that potentiated neurite outgrowth of PC12 cells. The methoxy functionality at C8 of the terminal sialic acid residue was essential for the activity. Furthermore, we showed that trisaccharide **4** stimulated neuritogenesis in human neuroblastoma SH-SY5Y cells via MAP kinase signaling. Trisaccharide **4** could promote neurite extension at low concentrations of up to 1 nM in a dose-dependent manner. We concluded that trisaccharide **4** is the active portion of the LLG-3 ganglioside that induces neuritogenesis in nerve cells via MAPK/ERK signaling, instead of Akt signaling.

The number of patients with neurodegenerative disorder, such as Parkinson’s disease and Alzheimer’s disease, continues to grow. Effective treatments for these diseases are urgently needed, and synthetic oligosaccharides of gangliosides may be useful compounds for treating neurodegenerative disorders or for regenerative medicine.

## Supplementary Files

Supplementary File 1
