# Supplementary material for: Structure-Activity Relationship Study of the Neuritogenic Potential of the Glycan of Starfish Ganglioside LLG-3
Source: Mar Drugs. 2015 Dec 5;13(12):7250–74. doi: 10.3390/md13127062 (PMC4699235; doi:10.3390/md13127062)
Supplement: Supplementary File 1 [file marinedrugs-13-07062-s001.docx]

**Supplementary Materials: Structure-Activity Relationship Study of the Neuritogenic Potential of the Glycan of Rev Starfish Ganglioside LLG-3 ^‡^**

Megumi Yamagishi, Ritsuko Hosoda-Yabe, Hideki Tamai, Miku Konishi, Akihiro Imamura, Hideharu Ishida, Tomio Yabe, Hiromune Ando and Makoto Kiso

**Figure S1.** Neurite outgrowth evaluation in PC-12 cells. Rat PC12 cells with the low serum culture medium with 1, 5, and 50 ng/mL of NGF were incubated for neurite outgrowth evaluation.
The error bar represents the standard deviation (S.D.).
